# Niche Differences in Coexisting Species: Ecological Insights Into the Role of Activity Patterns, Space Use, and Environmental Preferences

**DOI:** 10.1002/ece3.71802

**Published:** 2025-07-31

**Authors:** Carolina Reyes‐Puig, Urtzi Enriquez‐Urzelai, Neftalí Sillero, Antigoni Kaliontzopoulou

**Affiliations:** ^1^ CIBIO, Centro de Investigação em Biodiversidade e Recursos Genéticos, InBIO Laboratório Associado, Campus de Vairão Universidade do Porto Vairão Portugal; ^2^ BIOPOLIS Program in Genomics Biodiversity and Land Planning, CIBIO, Campus de Vairão Vairão Portugal; ^3^ Departamento de Biologia, Faculdade de Ciências Universidade do Porto Porto Portugal; ^4^ Instituto de Biodiversidad Tropical IBIOTROP, Museo de Zoología, Colegio de Ciencias Biológicas y Ambientales COCIBA Universidad San Francisco de Quito Quito Ecuador; ^5^ Czech Academy of Sciences Institute of Vertebrate Biology Brno Czech Republic; ^6^ CICGE‐Centro de Investigação em Ciências Geo‐Espaciais, Faculdade de Ciências da Universidade do Porto Vila Nova de Gaia Portugal; ^7^ Department of Evolutionary Biology, Ecology and Environmental Sciences, Biodiversity Research Institute (IRBio) Universitat de Barcelona Barcelona Catalonia Spain

**Keywords:** green lizards, microclimatic preferences, microhabitat use, species coexistence, temporal and spatial differences

## Abstract

The differences in niche structure enable the coexistence of ecologically similar species by reducing direct competition, combining spatial and temporal segregation with phenotypic variation that influences the differential use of resources and habitats. In this study, we examined niche differences in two coexisting green lizard species, *Timon lepidus* and *Lacerta schreiberi*, in northern Portugal. Both species differ in body size and habitat preferences, providing an excellent model to investigate mechanisms of niche differentiation. We integrated field observations on environmental variables (temperature, humidity, and illumination), body temperatures, microhabitat use, and activity patterns to complement previously published experimental results on mechanisms of species coexistence. Our observations revealed that *T. lepidus* prefers drier, rocky, and open areas and human‐built structures, while *L*. *schreiberi* was predominantly found in moist microhabitats with dense vegetation. Additionally, we detected differences in activity patterns, where *T. lepidus* displayed unimodal activity peaks around midday, while *L*. *schreiberi* exhibited bimodal patterns with activity peaks in the afternoon. These field‐derived patterns of microhabitat use and activity patterns were congruent with the thermal and water balance preferences of the two species quantified through previously published experimental trials. Spatial and temporal variations—particularly microhabitat relative humidity and time of activity—were found to be key factors in niche differences.

## Graphical abstract

This work provides novel insights into the spatial and temporal niche differences of two model species. Our findings reveal that microhabitat preferences and activity patterns are important factors of coexistence, complementing thermal and hydric regulation traits previously reported.

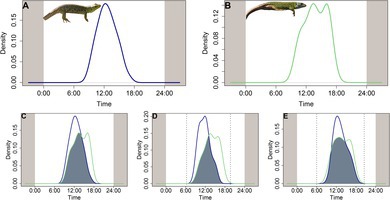

## 1 Introduction

For decades, the definition of the ecological niche has been a subject of debate among scientists (Grinnell 1917; Elton 1927; Hutchinson 1957, 1978; Soberon and Peterson 2005; Sales et al. 2021). From Hutchinson's seminal contribution in 1978 to the most current, biogeographically driven views of Soberon and Peterson in 2005, there is a widespread ecological niche definition that considers it a property of species, conceived as a multidimensional space. This multidimensional space encompasses the species' interactions with their environment and with other species, including the use of available resources, while factors such as dispersal patterns and life history traits influence how species occupy their niche. Together, this combination of factors shapes and defines the structure of the ecological niche (Sales et al. 2021). The ecological niche of a species comprises the fundamental niche, which includes abiotic, or scenopoetic, variables, and the realised niche, which is further moulded by biotic interactions with other species. Biotic interactions impose additional constraints, refining the niche beyond abiotic environmental conditions (Pearson 2007).

Ecologically similar coexisting species—those that live in the same area with similar requirements—may interact in three ways: through direct interference (individuals interact directly via aggression), exploitation‐competition (indirect competition for shared resources, which can lead to reduced availability of those resources), or by partitioning their niche (Pianka 1974, 2000; Begon et al. 1986; Dufour et al. 2015; Messerman et al. 2022). Niche differences refer to the ecological processes that allow coexisting species to reduce potential competition and occupy the same geographical area without displacing each other (Pianka and Huey 1978; Chapman and Reiss 1999; Valladares et al. 2015). To reduce competition, coexisting species often specialize in different resources. These resources can include microhabitats with specific structural and physical configurations, microclimatic conditions (e.g., thermal and hydric regulation), thermoregulation spots, and trophic resources. Indeed, co‐occurring species with generally similar environmental preferences may exhibit subtle differences in their particular ecophysiological requirements (e.g., selected temperatures, environmental humidity) (Díaz and Cabezas‐Díaz 2004; Reyes‐Puig et al. 2024). These differences not only influence their rates of development, growth, survival, and behavior but also enable them to exploit slightly different resources (Wolff 1996; Kearney and Porter 2009; Messerman et al. 2022).

On the other hand, the spatial distribution of coexisting species at local scales is a key factor in understanding niche differences (Dufour et al. 2015), as species may utilize different types of available microhabitats, such as varying amounts of vegetation, structural features, or water sources (Ganem et al. 2012; Meynard et al. 2012; Dufour et al. 2015). Similarly, temporal differentiation can be observed in the differential timing of foraging, breeding, and resting behaviors. This includes variations in daily and seasonal activity patterns, which are particularly relevant for investigating how species segregate their niche (Ganem et al. 2012; Meynard et al. 2012; Dufour et al. 2015). While studies addressing niche differences at the trophic level in coexisting species are abundant (MacArthur 1958; Kouete and Blackburn 2020; Kingsbury et al. 2020; Andriollo et al. 2021; Ramellini et al. 2024; Bezerra et al. 2024), other ways of reducing competition, including spatial (e.g., partitioning of space), environmental (e.g., differentiation in abiotic factors), temporal (e.g., activity levels or patterns) and, particularly, their combination is less explored overall. A key factor in understanding how different species modulate resource use, segregate their niches, reduce competition, and achieve coexistence is body size (Basset and Angelis 2007; Anaya‐Rojas et al. 2021; Reyes‐Puig et al. 2024). Body size influences dispersal capacity and resource utilization, enabling species of distinct sizes to exploit distinct resources, thereby reducing competition (Weil et al. 2023). Additionally, particularly in lizards, different phenotypic traits, such as head size and bite force, allow selective access to prey (Verwaijen et al. 2002; Huyghe et al. 2005), while limb length enables the use of different habitat structures (Irschick and Losos 1998). Since food resources are often limited by size (Prins and Olff 1998; Ritchie and Olff 1999), species with substantial differences in body size experience relaxed competition pressure when coexisting.

Due to notable differences in body size and ecophysiological parameters relevant for microhabitat use (Brito, Paulo, and Crespo 1998; Brito, Luís, et al. 1998; Marco 2017; Reyes‐Puig et al. 2024), *Timon lepidus* (ocellated lizard) and *Lacerta schreiberi* (Iberian emerald lizard) offer an outstanding model to explore niche differences and obtain insights into how microhabitat use, space partitioning, environmental preferences, activity patterns, and other components of the ecological niche are involved in species coexistence. Both species coexist in northern Portugal, allowing simultaneous observations, although they potentially exhibit differential use of microhabitats (Ferreira et al. 2016). *Timon lepidus* typically favors more open and semi‐arid areas (Ferreira et al. 2016; Renet et al. 2022), whereas *L*. *schreiberi* shows a stronger preference for humid environments with abundant surrounding vegetation (Salvador 1988; Brito, Paulo, and Crespo 1998; Brito, Luís, et al. 1998). Another relevant difference between the two species is in body size, where *T. lepidus* is quite larger (reaching a snout–vent length of 240 mm) than *L. schreiberi* (reaching a snout–vent length of 130 mm) (Brito, Luís, et al. 1998; Mateo 2017). The particular population studied in this research was selected because it represents one of the few well‐documented areas where both *T. lepidus* and *L. schreiberi* coexist in syntopy. Previous monitoring and available background data on the site provided an informed basis for studying the spatial and temporal dynamics of fine‐scale niches. Previous research suggested that body size differences between the two species may influence how they utilize environmental resources, leading to niche segregation (Reyes‐Puig et al. 2024). Indeed, based on laboratory experiments, we found that the primary driver of niche differences between both species is body size. However, these results also revealed that morphological and ecophysiological traits, independent of size, significantly contributed to such differentiation (Reyes‐Puig et al. 2024). Specifically, differences in niche were more pronounced on the water balance axis, with the combination of evaporative water loss and the effective proportion of the surface area that is wet. Likewise, the variance in preferred temperature contributed to the ecophysiological differentiation between *T. lepidus* and *L. schreiberi*. These hydric and thermal differences persisted even after controlling for body size, suggesting intrinsic physiological dissimilarities between both species (Reyes‐Puig et al. 2024). Based on these laboratory observations, we suggested that microhabitat differentiation may be a strong mechanism allowing the coexistence of these species in nature. Although there is some evidence that *T. lepidus* and *L*. *schreiberi* use microhabitats differently (e.g., Brito, Paulo, and Crespo 1998; Ferreira et al. 2016), further investigation is needed to understand how their body size and ecophysiological differences determine spatial and temporal niche differences in the field.

To fill this gap, here we integrate former knowledge on the physiology of these species from a field ecology perspective, to investigate real‐world consequences of previous experimental findings and better understand how they drive species coexistence and niche differences in nature. For this purpose, we explore activity patterns, humidity, and temperature in selected microhabitats, and inquire into their relationship with behaviour (type of activity in the field), microhabitat use, and fine‐scale distribution. If the physiological differentiation between ocellated and the Iberian emerald lizards, as derived from lab experiments, drives their capacity for segregation and niche differences, we expect to find no significant differences in body temperatures between species but significant differences in the relative humidity of their microhabitats. We also anticipate observing differentiation in space use and microhabitat composition, considering that fine‐scale spatial differences allow for resource partitioning and thus may reduce interspecific competition in coexisting species. We expect some degree of overlap in activity patterns, as species living in similar environments may have comparable thermoregulatory strategies, resulting in overlapping active periods to optimise conditions for basking or foraging (Žagar et al. 2015). However, we speculate there may be variations between species since seasonal activity patterns can vary in lizards (Adolph and Porter 1993). Also, we presume specific relationships between activity time, abiotic variables, and body size, where lizards with larger body sizes may require extended basking periods, starting earlier in the morning to achieve optimal body temperatures (Angilletta 2009); while smaller lizards may be active at differential time windows since their bodies reach optimal temperatures faster (Adolph and Porter 1993; Angilletta 2009). In terms of behavioural observations (i.e., activities such as basking, foraging and sheltering) and their relationship to environmental variables, we hypothesise that temperature and humidity differentiation will be influenced by the type of activity. Given the influence of landscape conformation on microhabitat availability, we expect to find a spatial distribution of species occurrences related to this landscape structure.

## 2 Material and Methods

### 2.1 Study Area, Model Organisms and Sampling Design

**FIGURE A1:**
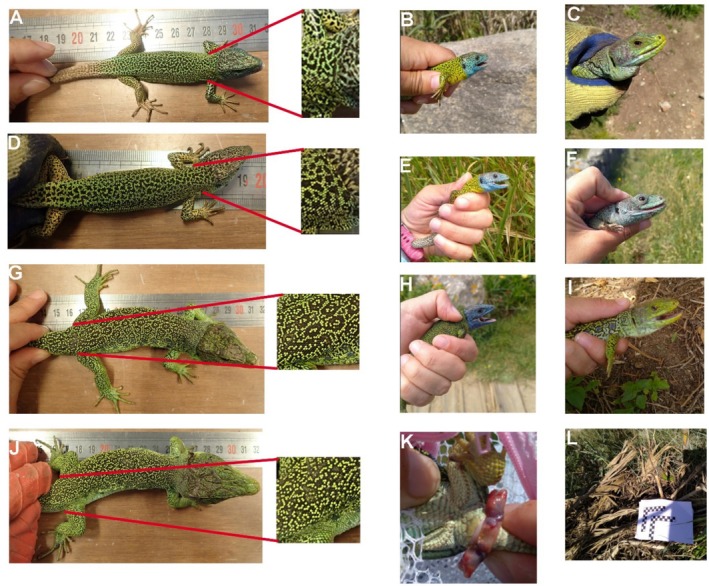
Representative photographs used for individual identification. (A, D, G, J) Dorsal views of individuals and zoomed‐in views of unique dorsal patterns. (B, C, E, F, H, I) Lateral views of head colouration, scale pattern, and morphological features aiding in sex determination and individual recognition. (K) Confirmation of sex by eversion of hemipenes in individuals lacking clear external sexual characteristics. (L) Habitat photo including a scale reference, used for body size estimation of non‐captured individuals.

**FIGURE A2:**
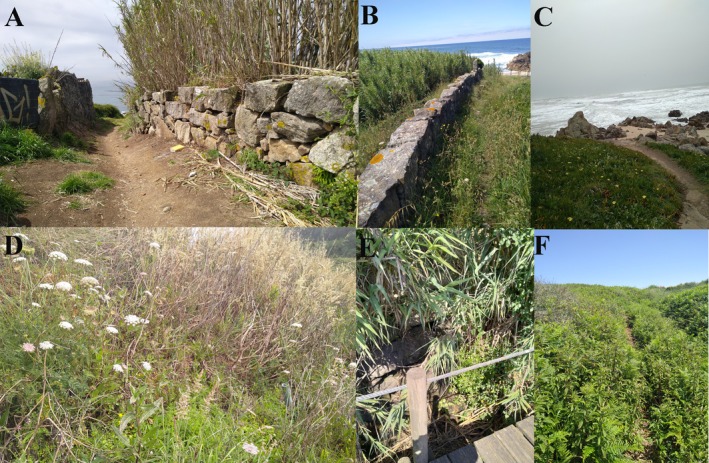
Microhabitats most commonly used by the two species of Mediterranean green lizards in Castro de São Paio. (A–C) *Timon lepidus*, (D–F) *Lacerta schreiberi*.

In this study, we focused on two species of green lizards distributed in the north of Portugal, *Timon lepidus* (Daudin 1802) and *Lacerta schreiberi* Bedriaga 1879. The ocellated lizard *T. lepidus* is typically found in western Mediterranean regions (Sillero et al. 2009) and it is distributed in the Iberian Peninsula, southern France, and Italy, ranging from sea level to 2000 m in elevation (Mateo and Cheylan 1997; Mateo 2017). In contrast, the Iberian emerald lizard *L*. *schreiberi* is an Atlantic species, endemic to the western Iberian Peninsula (Sillero et al. 2009), found from sea level to 2100 m in elevation (Brito, Paulo, and Crespo 1998; Marco and Pollo 1993). The most remarkable morphological characteristic that distinguishes both species is their body size, with the ocellated lizard being the largest green lizard in the Iberian Peninsula (reaching an SVL of 240 mm) and the Iberian emerald lizard the smallest green lizard in the region (up to 130 mm) (Brito, Paulo, and Crespo 1998; Marco 2017). While *T. lepidus* is recognized for its high ecological flexibility (Galán 2003; Llorente et al. 1995), *L*. *schreiberi* is closely associated with humid habitats such as moist deciduous forests and areas close to streams (Brito, Paulo, and Crespo 1998). In the period from January 2022 to mid‐July 2022, we sampled different areas and microhabitats in Castro de São Paio in northern Portugal (41.280297°N, 8.729283°W; 12 m asl, Figures A1 and A2), covering approximately 6 ha. This site hosts syntopic (co‐occurring) populations of *T. lepidus* and *L*. *schreiberi* are found. The landscape in Castro de São Paio comprises a diverse array of coastal features, including rocky shores, sandy beaches, and coastal vegetation (Araújo and Abrunhosa 2012). To document landscape heterogeneity, capture variation in microhabitat abundance, and investigate their relationship with environmental variables, lizard activity, space use, and behavior, we conducted random samplings throughout the study area, prioritizing the most abundant microhabitats (Figure A2): (i) herbaceous vegetation, areas with abundance of grasses, ferns, *Ulex* sp., *Carpobrotus* sp., and maize; (ii) rocky substrates, including exposed natural rocks without direct vegetation cover, stone walls following boundaries and delimiting agricultural zones in the study area, isolated rocks, or groups of rocks emerging from the ground; (iii) cement/rubble, encompassing areas with materials such as cement debris and bricks, generally derived from building structures, forming artificial substrates; (iv) walkway, that is, a wooden structure elevated above the ground, allowing safe passage of people across paths in the study area; (v) soil devoid of herbaceous vegetation, composed of mineral particles and organic matter. Sampling was conducted during the normal activity hours of the lizards, from 8:30 to 18:00, with one or two visits per week. We visited each microhabitat of the study area randomly, covering all available microhabitat both in the morning and afternoon, without sampling the same points within less than 4 h to ensure we obtained independent observations.

Once a green lizard was detected it was assigned a unique code, and its position was recorded by registering the exact geographic coordinates of observation using a sub‐meter precision GPS (Trimble GeoXT). All georeferenced points were subsequently cross‐checked and manually adjusted using a high‐resolution (< 1 m) orthophoto to ensure the correct identification of the microhabitat at the observation site. We also recorded the time, microhabitat type, air temperature and relative humidity (measured with a Lanaform LA 120701 Thermo‐Hygrometer), substrate temperature (measured with an GM320 infrared thermometer), and the lizard's behaviour. Air temperature and relative humidity were measured at the same height and position as the observed lizard to reflect the local conditions of the point of observation. We utilised the collected abiotic variables to characterise the microclimatic conditions of each microhabitat, providing context for the lizards' thermal environment and behaviour. We defined behaviour as the type of activity that each lizard engaged in at the moment it was observed and identified four types of behaviour: basking (immobile basking in the sun), foraging (actively searching for food and/or consuming it), sheltering (taking refuge in holes or hiding under the shade of vegetation or rocks), mating (interactions related to copulation), and fleeing (escaping from the proximity of humans). Similar classifications to define behaviour activities have been used (Diaz 1991; Pavey and Geiser 2008).

To avoid pseudoreplications, we visually observed the animals and took individual photographs with a camera, ensuring that each photo contained detail of the head and characteristic markings of each individual to later confirm their unique ID. An example of the photo‐identification method is provided in Appendix A: Figure A1. We photographed each individual and the surroundings of its point of observation, including a scale mark. A subset of individuals included in this study were previously involved in experimental procedures (Reyes‐Puig et al. 2024) and were marked with an apical tail clip to prevent recapture and reinforce individual identification. Whenever possible, we then collected lizards with a noose to measure their snout‐to‐vent length (SVL), and for those that could not be collected, we estimated body size from the scaled photographs. Sex was primarily assessed based on external features (e.g., head size, colouration, dorsal patterning). Individuals for which sex could not be confidently determined from morphology were captured and examined for the presence of hemipenes to ensure accurate sex identification (Appendix A). From May 2022 onwards, we extracted field body temperatures using a thermal camera (Cat S6Cat with an integrated FLIR TM Lepton camera), and we recorded the amount of light at the observation points with a light meter (ATP Electronics MT‐912). Additionally, to further minimize pseudoreplication, we avoided repeated sampling of the same spatial locations during each field trip. In cases where individuals were observed in close proximity (within 1 m), we only considered distinct if observed simultaneously, in order to avoid misidentifying a single moving individual.

### 2.2 Data Analyses

#### 2.2.1 Environmental Variables and Microhabitat Preferences

To describe patterns of microhabitat use by the two species, we recorded the number of field observations of *T. lepidus* and *L. schreiberi* in each microhabitat within the study area. Given the likely spatial correlation between observations, we did not apply statistical tests that assume independence or equal use as a null expectation. Instead, we reported the observed frequencies as descriptive data to illustrate the trends in habitat selection for each species.

To identify differences between species in air temperature, substrate microhabitat temperature, and relative humidity of the points of observation, as well as illuminance and body temperature, we used analyses of variance (ANOVA), including these environmental parameters as dependent variables and species, sex, and their interaction as predictors. We considered only adults, as we had very few juvenile observations. As the effect of sex was not significant for any of the above analyses, we did not consider it a factor of interest for subsequent analyses. To investigate whether differences between species in air temperature, substrate microhabitat temperature, relative humidity, illuminance, and body temperature depended on the type of microhabitat where lizards were observed, we performed an ANOVA with species, microhabitat, and their interaction as factors. Note that, as we did not record observations of *L. schreiberi* in the walkway, this microhabitat category was removed from the ANOVA. To investigate whether variation in the recorded variables depended on the combined effects of species and type of behaviour, we performed an ANOVA with these factors and their interaction as predictors.

**FIGURE 1:**
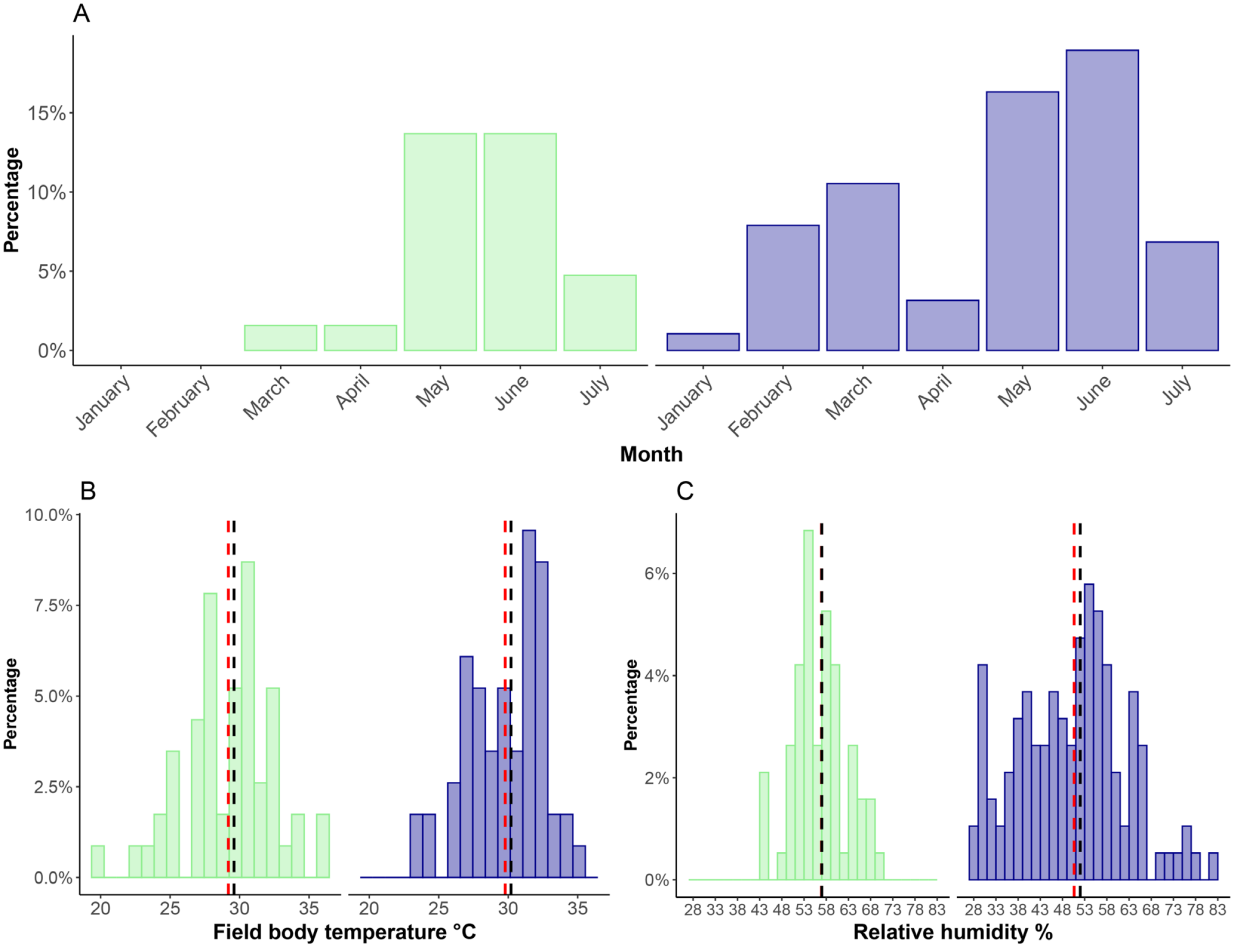
Percentage of observations during the sampling period (A), field body temperatures (B) and relative humidity (C) for *T. lepidus* (blue) and *L. schreiberi* (green) during the entire sampling period. Bars represent the percentage of individuals per bin. Red dashed and dotted lines indicate the mean for *T. lepidus* and *L. schreiberi*, respectively; black dashed and dotted lines indicate the corresponding medians. 100% of the observations in (B) and (C) correspond to the sum of all bins.

Finally, we explored seasonal variation across species in the recorded traits. As we recorded very scarce observations of *L. schreberi* during the winter, and the observations during March and April were very few (Figure 1), we only considered the data from spring and summer (from May to July) for this analysis, and we used an ANOVA to test for variation in air temperature, substrate microhabitat temperature, relative humidity, illuminance, and body temperature in response to species, month, and their interaction. We assessed the significance of all ANOVAs via residual randomisation procedures with 1000 permutations using the package “RRPP” (Collyer and Adams 2019) in R (R Core Team 2024), a non‐parametric approach particularly suitable for data sets where traditional assumptions may not be fully met. We performed post hoc pairwise comparisons whenever a significant effect or interaction was identified in ANOVAs involving factors with more than two categories with the function “pairwise” of the same package.

#### 2.2.2 Activity Patterns

We used independent lizard observations to assess species' seasonal distribution (i.e., activity patterns) and proportion of the daily cycle that a species is active (i.e., activity levels). We first evaluated differences in levels of activity between species for the complete data set, i.e., including all observations from January through July. We then evaluated differences between and within species in activity patterns. For this purpose, observations were grouped into seasons based on solstice limits for the Northern Hemisphere, i.e., divided into winter (January, February, and up to 21 March), spring (22 March–21 June), and summer (22 June–31 July). We considered only spring and summer months for interspecific comparisons, as winter observations were very scarce for *L*. *schreiberi*.

We conducted activity analyses using the ‘activity’ package (Rowcliffe 2023) in R version 4.3.3 (R Core Team 2024). First, we converted dates and times to radians and calculated the circular mean for each species (i.e., the mean direction of activity hours expressed as angles across a 24‐h cycle, in radians). We then used the “fitact” function with 9999 replicates to fit kernel density functions that describe lizard activity.

We compared daily activity between the two species, using the Wald test with the function “compareAct”, and compared and identified differences in seasonal activity patterns using the function “compareCkern” with 9999 replicates, which tests for the probability that two circular observations come from the same distribution. In order to assess the degree of temporal niche overlap, we estimated the overlap coefficient (Δ) between *T. lepidus* and *L*. *schreiberi* patterns, where Δ = 0 reflects no overlap and Δ = 1 indicates total overlap (Ridout and Linkie 2009). We used Δ1 as recommended by Ridout and Linkie (2009) and Meredith et al. (2024) for small sample sizes (*n* < 70), which provide lower root mean square error (RMSE) when the sample size is small. We used the “overlap” package (Meredith et al. 2024) for overlap estimations and for producing kernel density plots of lizard activity with the “overlapPlot” function. It is important to note that, although we implemented several strategies to minimize individual autocorrelation, the analytical approach used here does not require that each observation represent a unique individual. The method estimates activity patterns from independent records, meaning detections that are assumed to be uncorrelated in time (Rowcliffe et al. 2014). We considered independent records as those observations that were collected at least 4 h apart within each location, ensuring temporal independence of sampling events.

To evaluate whether activity times are influenced by environmental variables, body temperatures and body size, we performed Bayesian circular GLMs, since time follows a circular distribution (Lee 2010; Ridout and Linkie 2009). We constructed a set of candidate models including biologically relevant predictors of lizard activity (e.g., SVL, air temperature, relative humidity, body temperature, illuminance, and species identity), and their interactions. We used the package “circglmbayes” (Mulder and Klugkist 2017) which allows regressing a circular variable on linear and categorical predictors. We used the “circGLM” function, which implements a Markov chain‐based Monte Carlo (MCMC) approach. We estimated the coefficients, standard deviations (SD) and 95% credible intervals of the estimated coefficients. The credible intervals (lower bound, LB, and upper bound, UB) were obtained from the posterior distribution of the coefficients, and we considered a relationship to be significant if the credible intervals did not include the value 0, as this would indicate no effect (Hespanhol et al. 2019). We ran the models with 9999 iterations. To assess model predictions, we compared the values of the Deviance Information Criterion (DIC) and the Watanabe‐Akaike Information Criterion (WAIC). To evaluate the convergence of the model we used the “coda” package (Plummer et al. 2006) and the “gelman.diag” function, which calculates the Gelman and Rubin convergence diagnostic. Convergence diagnostics ascertain whether the MCMCs have adequately explored the parameters and converged to a stable distribution. The Gelman and Rubin diagnostic compares the within‐chain variance with the between‐chain variance. Convergence is indicated by $\hat{R}$ values, where values closer to 1 denote good convergence. Model selection was based on the comparisons of DIC and WAIC, and we favoured the model with the best balance between goodness of fit and less parameters (i.e., parsimony).

#### 2.2.3 Micro‐Scale Distribution

To compare how species are distributed locally (i.e., in the same population) at a microscale, we first obtained an orthophoto to spatially record the occurrences of the two species in the study area. Occurrence data were imported into ArcGIS Pro (ESRI 2024) and projected to the WGS 1984 UTM Zone 29 N coordinate system. We then plotted the occurrence points on the map to visualize the spatial distribution of the species. To identify hotspots of occurrence for each species and relate them to potential spatial differences, we used Kernel density estimation (KDE) implemented in ArcGIS Pro 10.x (ESRI 2024). This technique smooths discrete location data to generate a continuous estimate of the density of occurrence points (ESRI 2024). We chose KDE because it effectively captures the intensity of occurrence points across space, providing a clear visualization of data presence. Moreover, it does not assume an underlying distribution of the data (Baíllo and Chacón 2021). We identified the hotspots based on densities that fall within the 75th percentile of the Kernel Density Estimation (KDE) output, representing areas where 75% of the highest density aggregations are located.

## 3 Results

We recorded a total of 206 lizard observations with concomitant standardized records of different field variables (body temperature, microhabitat substrate temperature, air temperature, relative humidity, illuminance, behaviour, microhabitat type, month and hour). Of these observations, 134 were of *Timon lepidus* (♂ = 83, ♀ = 44, juveniles = 7), and 79 of *Lacerta schreiberi* (♂ = 53, ♀ = 24, juveniles = 2). Observations of *T. lepidus* occurred from January to the end of the sampling period in July; the months with the most observations were February, March, May, and June (Table A1, Figure 1A). On the other hand, we did not record individuals of *L. schreiberi* during January and February, but only from March until July, the months with the highest number of observations being May and June (Table A1, Figure 1A). *Timon lepidus* generally exhibited greater variation in relative humidity, with peaks in winter and spring, while *L. schreiberi* had higher average values and a narrower range (Figure 1A–C, ANOVA on relative humidity, *Z* = 3.415, *p* < 0.001).

### 3.1 Microhabitat Use and Microclimatic Preferences

Mean values for all variables, factors, and the numbers of observations are available in Table A1. We did not detect significant differences in air temperature, illuminance, field body temperature, or microhabitat substrate temperature at the observation points of *T. lepidus* and *L. schreiberi*, nor between sexes (Table A2). However, we found significant differences in relative humidity between species but not between sexes (Table A2).

**FIGURE 2:**
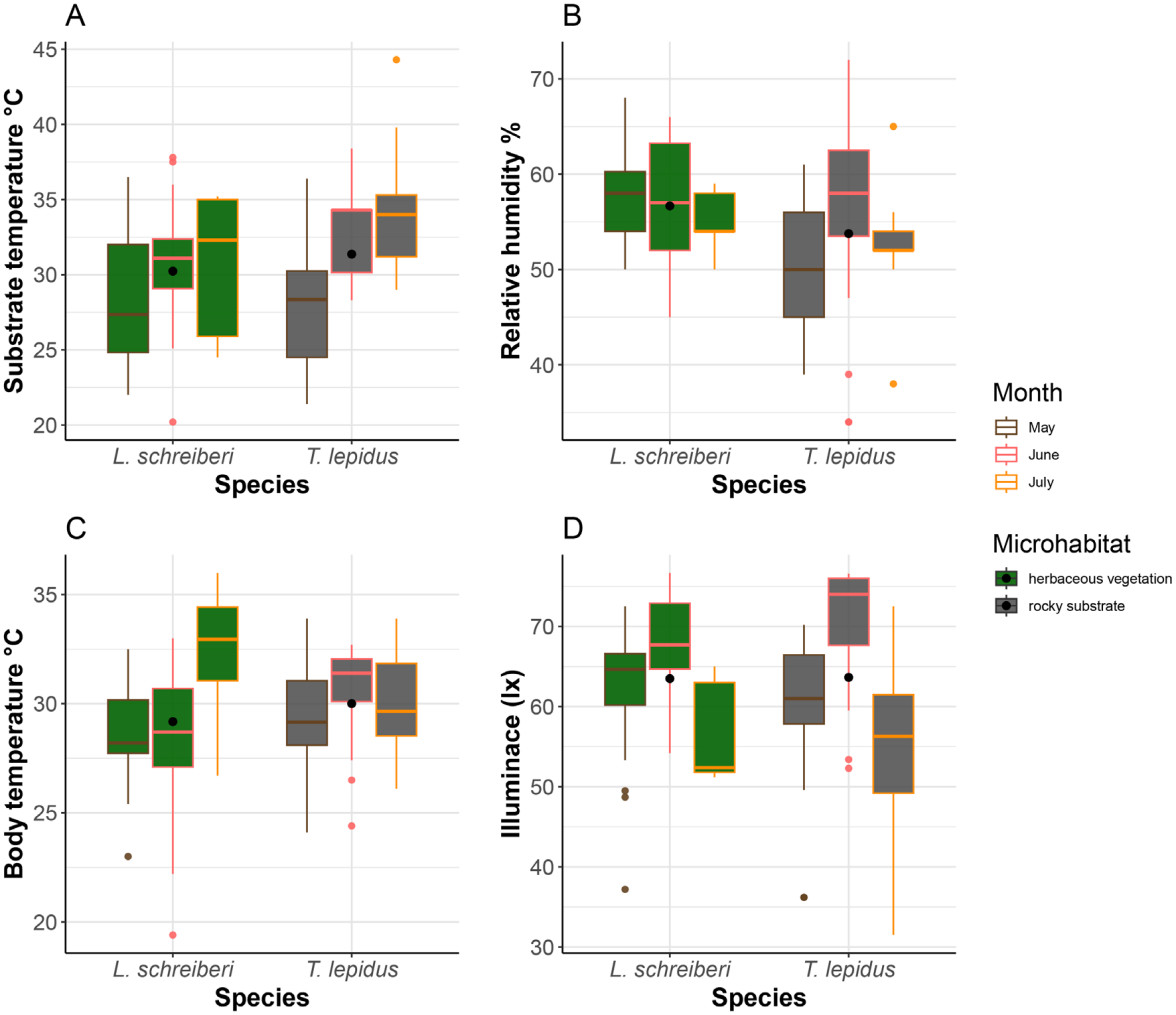
Substrate microhabitat temperature (A), relative humidity (B), body temperature (C), and illuminance (D) for *T. lepidus* and *L. schreiberi* in herbaceous vegetation and rocky substrate across different months (May–July).

We found a great variation in the frequency of using different microhabitats for *T. lepidus* and *L*. *schreiberi*. *T. lepidus* predominantly used rocky substrate (70.1%), followed by cement/brick (12.7%), herbaceous vegetation (9.7%), bare soil (3.7%), and walkway (3.7%). In contrast, *L*. *schreiberi* mainly used herbaceous vegetation (86.4%), with lower proportions of cement/brick (5.1%), rocky substrate (3.4%), bare soil (3.4%), and walkway (1.7%). We detected a significant effect of microhabitat on air temperature and a significant species–microhabitat interaction on illuminance (Table A3). Pairwise analyses confirmed differences in illuminance (Table A4). For *T. lepidus* , substrate temperature in rocky microhabitats varied significantly across months (*Z* = 3.999, *p* = 0.001, Figure 2A), while relative humidity showed marginal differences (*Z* = 1.636, *p* = 0.057, Figure 2B), and illuminance differed significantly (*Z* = 3.448, *p* = 0.001, Figure 2D: Appendices B, C, D).

Field body temperature did not vary across months in rocky microhabitats (*Z* = 0.525, *p* = 0.299, Figure 2C). For *L*. *schreiberi*, no monthly differences were detected in substrate temperature (*Z* = 0.767, *p* = 0.241, Figure 2A) or relative humidity (*Z* = 0.051, *p* = 0.488, Figure 2B) in herbaceous vegetation. However, body temperature (*Z* = 2.145, *p* = 0.017, Figure 2C) and illuminance (*Z* = 2.949, *p* = 0.001, Figure 2D) showed significant monthly variation. Microhabitat substrate temperatures were similar between species (Figure 2A, Table A3). By contrast, body temperatures peaked in July for *L. schreiberi* and in June for *T. lepidus* (Table A5).

**FIGURE 3:**
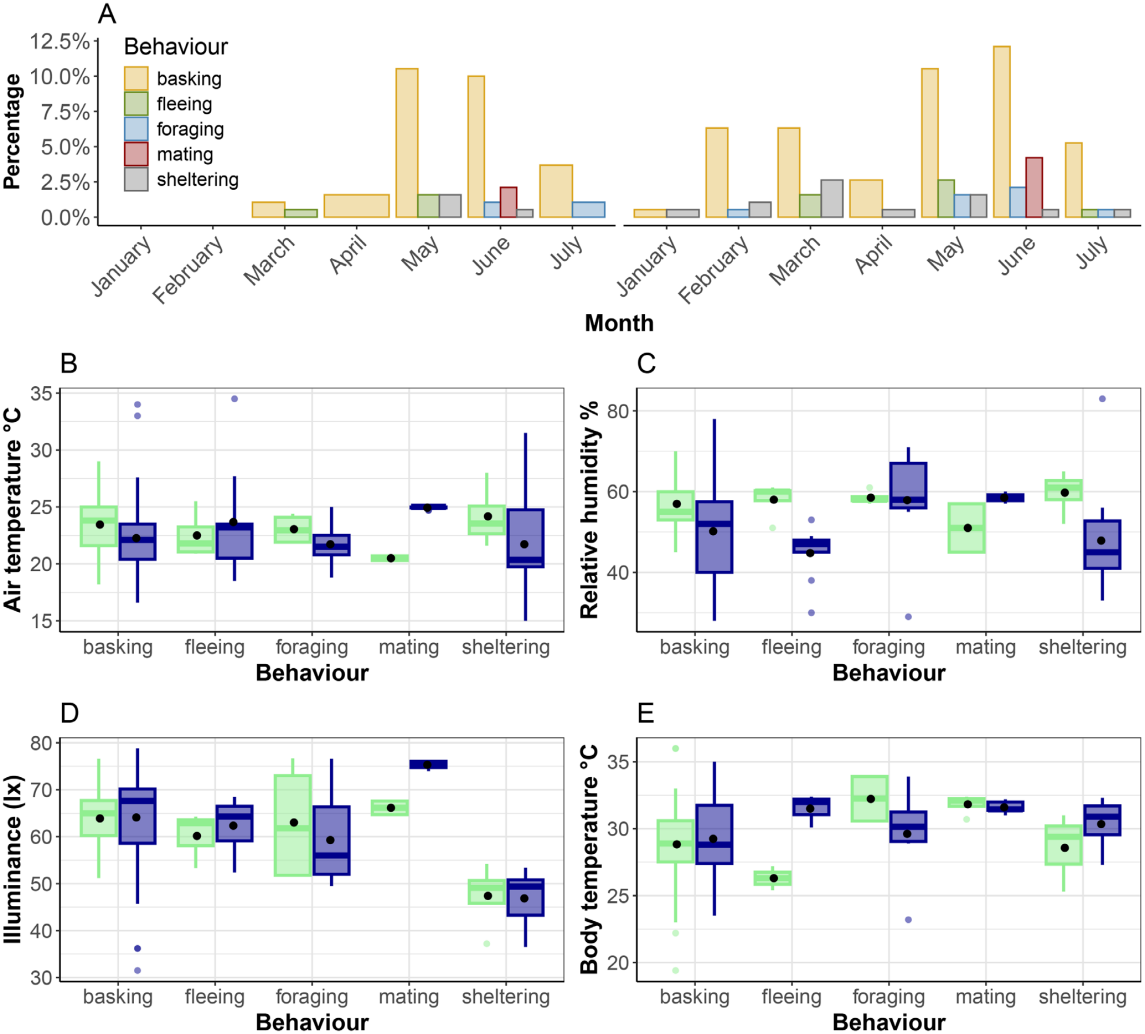
Behaviour and environmental conditions of *T. lepidus* and *L. schreiberi* over time. Percentage distribution of behaviours observed for. *T. lepidus* (right) and *L. schreiberi* (left) from January to July (A). Behaviours include basking, fleeing, foraging, mating, and sheltering. Air temperature (B), relative humidity (C), illuminance (D), and body temperature associated with each behaviour (E). The blue and green boxplots represent *T. lepidus* and *L. schreiberi*, respectively.

We detected a significant difference depending on behavior for illuminance, field body temperature, and microhabitat substrate temperature (Table A6). Additionally, we found a significant interaction between species and behavior for air temperature (Table A6). Most green lizards in Castro de São Paio were observed basking, with *T. lepidus* showing the highest activity in February, March, May, and June, and *L. schreiberi* in May, June, and July (Figure 3A). We observed mating in June for both species (Figure 3A) and detected significant differences in air temperature during mating between species (Figure 3B, Table A7). We did not detect a significant effect of behavior on relative humidity (Table A6, Figure 3C). Illuminance was highest during mating and lowest during sheltering (Figure 3D), although pairwise analyses found no significant differences (Table A8). Finally, observations of body temperature and microhabitat substrate temperature were heterogeneous, with no significant pairwise differences (Figure 3E, Tables A9 and A10).

**FIGURE 4:**
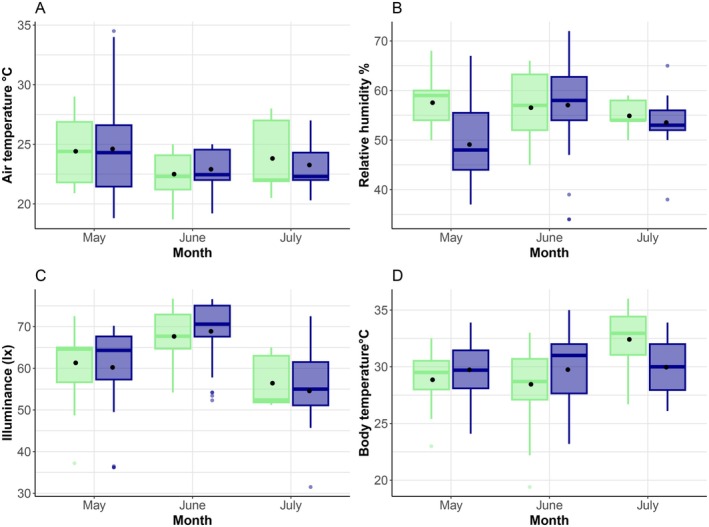
Boxplots of variables during the sampling period related to the observations of *T. lepidus* (blue) and *L. schreiberi* (green) and air temperature (A), relative humidity (B), illuminance (C), and body temperature (D).

**FIGURE A3:**
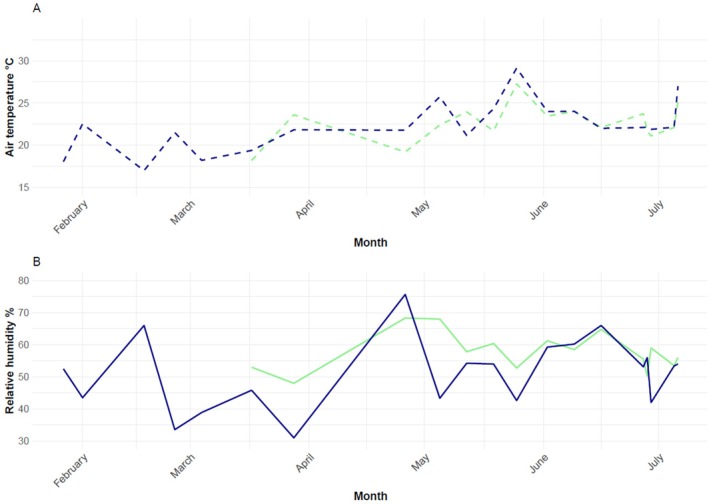
Mean air temperature (dashed lines) (A) and mean relative humidity (solid lines) (B) during the sampling period.

Seasonal variation in air temperature showed lower values in winter and spring, and humidity peaking between February and May (Figure A3). We detected significant monthly differences in air temperature (Table A11), although the subsequent pairwise comparisons were not significant (Table A12). When analyzing relative humidity during months with higher observations (May, June, July), we found a significant effect of species, months, and their interaction (Table A11). Significant differences occurred in May, with *T. lepidus* showing lower values than *L. schreiberi* (Figure 4B, Table A13). A similar pattern was observed in March, although not statistically tested. We also found significant monthly variation in illuminance (Table A11, Figure 4C), although pairwise analyses showed no differences (Table A14). Field body temperature varied significantly across months, with differences mainly occurring in June and July (Figure 4D, Tables A5 and A11).

### 3.2 Activity Patterns

**FIGURE 5:**
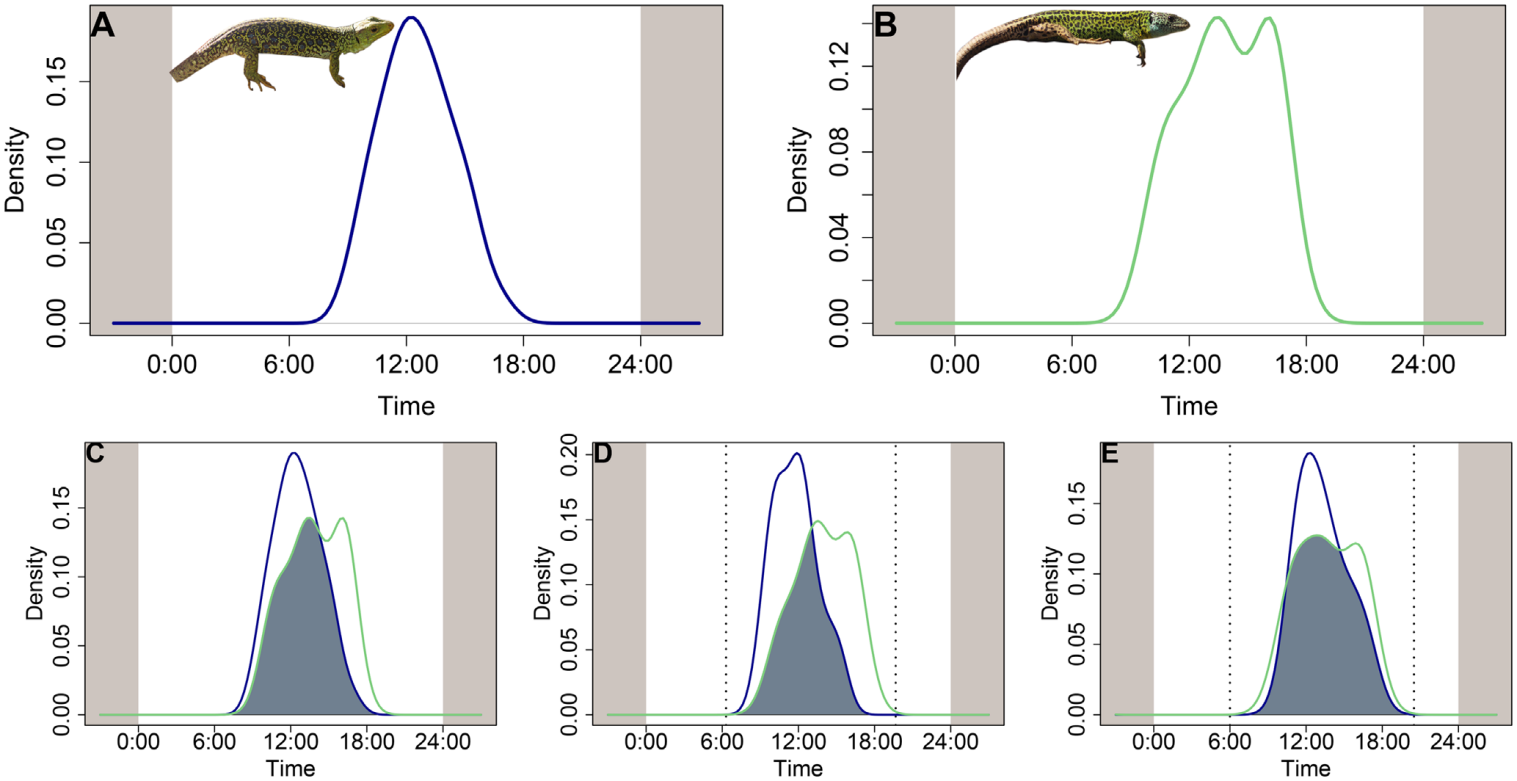
Activity patterns of *Timon lepidus* (blue) (A) and *Lacerta schreiberi* (green) (B), and overlap (blue‐grey shaded area) between species for general activity patterns (C), spring activity patterns (D), and summer activity patterns (E).

Our observations indicate that *T. lepidus* exhibits a unimodal activity pattern with a pronounced peak around midday (Figure 5A), a trend that was maintained through all three seasons (winter, spring and summer) (Figure 5). On the other hand, *L. schreiberi* showed a bimodal pattern with activity concentrated mainly at 2 h past noon between 1:00 and 2:00 p.m., and another peak in the afternoon mainly between 5 and 6 p.m. (Figure 5B). This pattern was similar in the spring and summer data (Figure 5A).

Regarding activity levels (i.e., the proportion of the daily cycle that the species is active), we detected significant differences between the two species, with *T. lepidus* being more active (*W* = 4.74, *p* = 0.030). On the other hand, the distribution of the activity patterns of both species differed significantly (*p* < 0.001), despite a relatively high overlap coefficient (Δ = 0.76, 95% CI = 0.65–0.97, Figure 5). When we analyzed the spring dataset, we observed that the overlap between species in activity patterns was the most reduced, with significant differences between the patterns of *T. lepidus* and *L*. *schreiberi* (Δ = 0.59, 95% CI = 0.44–0.70, *p* < 0.001, Figure 5D). By contrast, the summer dataset exhibited the highest overlap coefficient, and activity pattern distributions did not differ significantly (Δ = 0.84, 95% CI = 0.69–0.99, *p* = 0.275, Figure 5E).

Our Bayesian circular GLMs showed that the models that best explain differences in activity times were those that included environmental temperature (Table A15). We detected significant differences in activity times between species: we found a significant interaction between air temperature and species *T. lepidus* (Table A15) suggesting that *T. lepidus* tends to be active earlier in the day as air temperature increases. We did not detect significant interactions between body size and other variables (air temperature, relative humidity, body temperature and illuminance) in the best models explaining activity time (Table A15). It is important to note that of the 206 total field observations, SVL could be assessed in 146 cases: 126 individuals were directly measured after capture, and for 20 individuals, SVL was estimated from scaled field photographs taken under standardised conditions (Figure A1).

### 3.3 Spatial Distribution of Species

**FIGURE 6:**
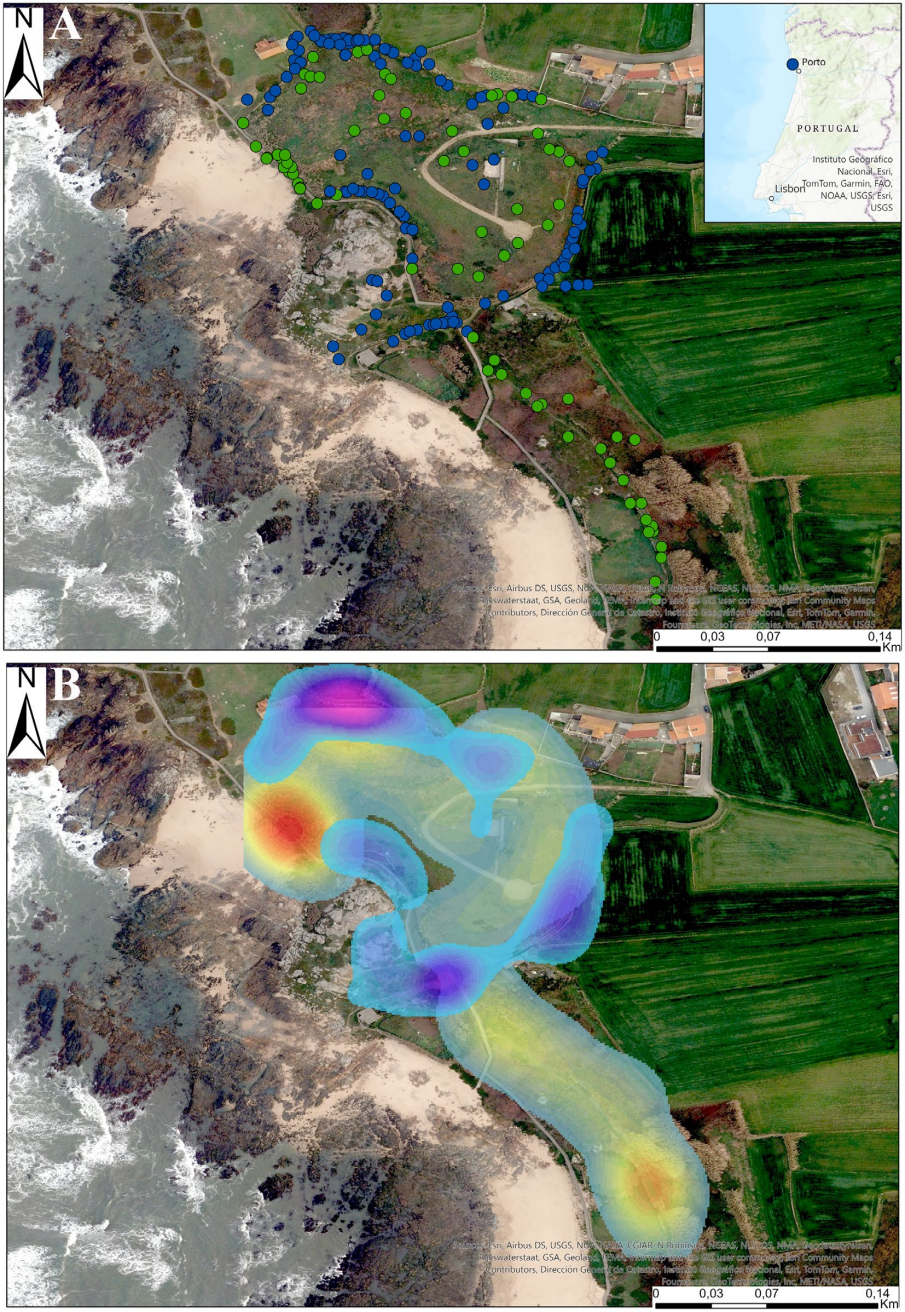
Spatial distribution and density of observations of *T. lepidus* and *L. schreiberi* in the study area. (A) Distribution of observation points for *T. lepidus* (blue points) and *L. schreiberi* (green points) along the coastal and inland areas in Castro de São Paio. The inset map shows the location of the study area near Porto, Portugal. (B) Kernel density estimation (KDE) map showing the density of occurrences for both species. Areas with higher densities are indicated by warmer colours, red and yellow for *L. schreiberi* and blue and purple for *T. lepidus* .

Our observations of nearly 7 months of sampling allowed us to pinpoint spatial hot spots where sightings of each species were concentrated. *T. lepidus* was predominantly detected in open areas and along human‐accessible pathways (edges of walkways, abandoned structures with debris, etc.). Observation points indicate that *T. lepidus* could be found around rocky edges and natural rock strata, including areas with vegetation or along coastal sandy substrates (Figure 6, Figure A2). In contrast, *L. schreiberi* was observed in areas covered by herbaceous vegetation (Figure 6, Figure A2). While some overlap exists in the spatial distribution of observations, distinct areas of occurrence were evident between the species. For *T. lepidus* , we identified at least three high‐density hot spots in the study area, all closely associated with rocky microhabitats (Figure 6). Meanwhile, *L. schreiberi* exhibits at least two high‐density hot spots: one associated with a small stream flowing towards the coast and another in areas densely vegetated with shrubs and herbaceous plants (Figure 6, Figure A2).

## 4 Discussion

Niche differences among co‐occurring species remain a critical point of debate in current ecology (Amarasekare 2002; Gravel et al. 2011; Valladares et al. 2015). Experimental evidence and empirical field observations are essential to thoroughly understand the mechanisms by which ecologically similar species can thrive in the same localities. Our field study of green lizards in northern Portugal revealed significant differences in microhabitat use and preferred conditions of relative humidity between *T. lepidus* and *L. schreiberi*, which appear to contribute to their niche differences. Specifically, *L*. *schreiberi* preferred relatively humid microhabitats, and it followed a bimodal activity pattern, whereas *T. lepidus* used drier, more exposed habitats, and it exhibited a peak of daily activity fitting a unimodal pattern. These findings suggest that physiological, ecological, temporal, and spatial factors—including microhabitat humidity, spatial arrangement, and activity patterns—contribute to observed niche differences between the two species.

During the sampling period, which encompassed the months of highest activity and the reproductive period of both species, we obtained a higher number of observations for *T. lepidus* than for *L*. *schreiberi*. This may be the result of a combination of various factors, including detectability (MacKenzie et al. 2017). Field observations indicated that *L*. *schreiberi* prefers microhabitats with dense herbaceous vegetation over rocky or open areas, consistent with findings from other researchers (Brito, Paulo, and Crespo 1998; Brito et al. 1999). This suggests that although *L. schreiberi* may occasionally use other types of microhabitats such as stone walls (Brito, Paulo, and Crespo 1998), it shows a preference for herbaceous and bushy vegetation (Brito, Paulo, and Crespo 1998; Brito, Luís, et al. 1998; Brito et al. 1999), which may reduce our potential for observing it. Although our sampling covered all microhabitats present in Castro de São Paio, factors such as vegetation height, the noise produced by the researchers during sampling, the lizards' size and cryptic coloration, and the ease of access to certain areas may have directly reduced detectability (MacKenzie et al. 2017; Driscoll et al. 2020). Nonetheless, considering that we sampled the entire study area across different months, we identified that *L. schreiberi* is less observable during the winter months, potentially due to its reduced activity during this period (Marco and Pérez‐Mellado 1998; Pérez‐Mellado 1998). On the other hand, *T. lepidus* was detectable in the sampled period during winter, although in a smaller proportion than in the spring and summer, which implies that this species could be more adaptable to seasonal variations in temperature and rainfall (Figure A3). Previous studies have shown that a reduction of lacertid activity during the autumn and winter is related to the preparation for the breeding period and the accumulation of lipid reserves (Carretero 2006; Sannolo et al. 2019). Given its larger body size and associated greater thermal inertia, *T. lepidus* is expected to be more active during winter, as it can maintain more stable body temperatures even amid extreme thermal fluctuations (Stevenson 1985). This is consistent with our observations, where *L. schreiberi*, significantly smaller than *T. lepidus* , was scarcely detected during the winter and early spring months.

**FIGURE A4:**
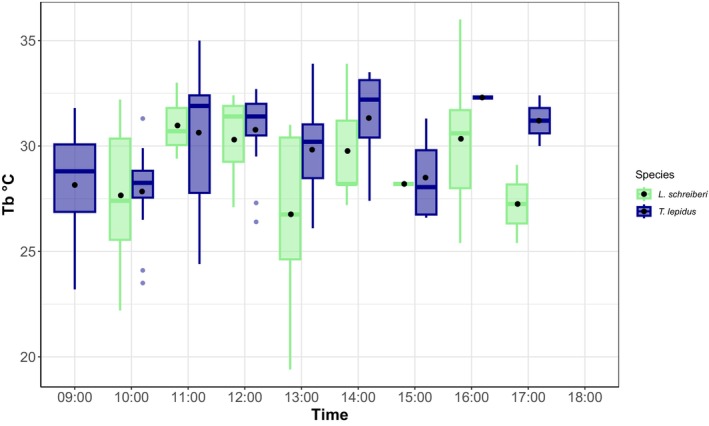
Field body Temperature (Tb) of *L. schreiberi* (green) and *T. lepidus* (blue). Each box represents the interquartile range with the median value indicated by a thick horizontal line inside the box. Outliers are shown as individual points. The black dots represent the mean body temperatures for each time period.

Our findings suggest that air temperature, illuminance, and field body temperature do not contribute to niche differences between the two species. Although these variables exhibit seasonal variation (Salvador 1988; Brito et al. 1999), they do so similarly in both *T. lepidus* and *L*. *schreiberi* (Table A11). These field data corroborate laboratory experiments on thermal ecology with the same populations, where no differences were detected in the preferred temperature of the two species (Reyes‐Puig et al. 2024). Additionally, our results show no differences in these variables between the sexes, which suggests that both sexes of the two species have a similar ability to regulate their temperature within the thermal ranges available in their environment. However, it is well established that females may exhibit differential thermoregulatory behavior during periods of intensified physiological demand, such as oogenesis and embryogenesis (Shine 2004; Lailvaux 2007). While our study did not specifically focus on these reproductive periods, variations in body temperatures between sexes may likely occur before and after the breeding period. Although our results showed that *T. lepidus* and *L*. *schreiberi* use different microhabitats, it seems that these can provide similar thermal conditions allowing both species to reach comparable body temperatures (Figure 2). This observation is supported by the fact that both species thermoregulate on average at similar temperatures, both experimentally (Reyes‐Puig et al. 2024) and in nature (see Figure A4). Since thermal conditions alone do not appear to significantly contribute to the observed niche differences between two species, it is likely that the spatial configuration of microhabitats, combined with microclimatic conditions, plays a more influential role in driving spatial divergence (Prieto‐Ramirez et al. 2018). Such a hypothesis still needs to be corroborated, though, potentially through fine‐scale microclimatic modeling of the habitats used by the two species.

Another key factor which could influence niche differences, but that unfortunately has been largely overlooked in squamate ecophysiology, is water balance (Kearney et al. 2018). Our results, in line with other research (Brito et al. 1999; Ferreira et al. 2016; Reyes‐Puig et al. 2024), underscore the importance of water balance traits for *L*. *schreiberi* and for the coexistence of the two species. The relative humidity of the microhabitats where we observed individuals of this species was significantly higher than that of the microhabitats used by *T. lepidus* , which tend to be open, stony, and more exposed, similar to findings reported by other authors (Mateo 2017; Mateo, Escoriza and Amat 2021). Herbaceous and shrub vegetation store more moisture and receive less radiation, reducing direct evaporation and increasing microenvironmental and body temperatures (Adams 2009). Consequently, organisms occurring in highly humid habitats may face increased water loss during periods of elevated temperatures, as these conditions can intensify evaporative stress (How and Lee 2014; Weaver et al. 2023). Consistent with our field findings, *L. schreiberi* not only uses wetter sites in the landscape but specifically prefers areas near streams with flowing or semi‐flowing water, as noted by several other researchers (Salvador 1988; Pérez‐Mellado 1998; Brito et al. 1999). Recently published evidence (Reyes‐Puig et al. 2024) confirms that the Iberian Emerald lizard has higher water loss rates than *T. lepidus* and also holds a higher proportion of wet skin surface. Skin permeability appears to be a crucial trait for coping with physiological changes associated with water loss (Sannolo et al. 2018; Weaver et al. 2023), which may explain why *L*. *schreiberi* needs to be near bodies of water and humid places, to avoid higher desiccation rates, as observed in other organisms (Cain et al. 2006; Weaver et al. 2023). Likewise, laboratory experiments have identified that skin water loss in lizards is greater in hot and humid environments. This phenomenon may be related to vapour pressure and the amount of water in the environment (Weaver et al. 2023). However, reptiles from humid habitats tend to exhibit lower resistance to skin water loss, reflecting a lower selective pressure for water retention compared to species from arid environments (Dmi'el 2001).

In the context of observed niche differences among coexisting species, behavior plays a crucial role, especially when thermoregulatory strategies are closely tied to it (Díaz and Cabezas‐Díaz 2004). Furthermore, humidity is not only relevant for adult physiology, but may also influence reproductive success by directly affecting the development of eggs, which are known to be very sensitive to desiccation. Thermal and hydric constraints during embryonic development in oviparous organisms cannot be regulated by behavioral traits, so external temperature and humidity play a critical role in hatchling survival (Kearney and Enriquez‐Urzelai 2023). The type of behavior or activity is directly related to the internal metabolism of the species (Angilletta 2009). For instance, when ectotherms mate, body temperature increases mainly because organisms seek out exposed sites with higher radiation to copulate (Licht 1972; Angilletta 2009). Our observations corroborate this, as body temperatures were higher for both species when mating compared to basking, which was also associated with higher illuminance in the exposed sites (Figure 3). Likewise, when we analyzed our data by type of behavior, we found that *L*. *schreiberi* had higher body temperatures while foraging compared to other activities. Although both species are considered active foragers, this could suggest that *L*. *schreiberi* requires a higher energetic output or adopts distinct thermoregulatory strategies during foraging. In this sense, trophic niche differentiation is likely influenced not only by dietary differences (Kaliontzopoulou et al. unpublished data) but also by the energy demands and thermoregulatory behaviors associated with foraging mode (Bowker 1984; Angilletta 2009).

Within the landscape arrangement of the study area (Figure 6), the spatial organization of microhabitats is shaped by structural features and human accessibility. Castro de São Paio is a highly frequented touristic site (Araújo and Abrunhosa 2012) where anthropogenic structures like rocky edges bordering agricultural zones, as well as open, vegetation‐free areas such as coastal rocks, provide ideal conditions for observing *T. lepidus* (Figure 3, Figure A2). Conversely, *L. schreiberi* is more restricted to areas covered by herbaceous vegetation, often with limited human accessibility, dominated by ferns and grasslands within the study site (Figure 6). Spatial aggregations of both species in distinct areas reflect that spatial segregation is an important driver of niche differentiation, as pointed out by several authors (Darmon et al. 2012; Dufour et al. 2015; Hart et al. 2017).

On the other hand, temporal niche differences allow coexisting species to access exclusive resources, such as food and space. Therefore, understanding the timing of activities and periods of peak activity provides valuable information on how species potentially segregate their niches. Our results support the idea that *T. lepidus* and *L*. *schreiberi* differ in their ecological niches not only through phenotypic differentiation—primarily due to divergence in body size, which in turn leads to differences in bite force, maneuverability, and physiological traits (Reyes‐Puig et al. 2024), but also spatially and temporally. While both species are diurnal, they exhibited noticeable differences in their activity peaks. *Timon lepidus* exhibits a clear unimodal peak around midday, whereas *L*. *schreiberi* is more active after noon, particularly between 5 and 6 p.m. Although we observed more activity of *T. lepidus* compared to *L. schreiberi* during cooler months, we also detected that its diurnal peak coincides with the hottest hours of the day, which may suggest that its body size and associated thermal inertia may allow it to function efficiently under both seasonal temperatures and diurnal thermal conditions.

There is limited detailed information on the activity patterns of both species. Previous studies have reported temporal activity ranges primarily during autumn (Sannolo et al. 2019) and spring (e.g., Marco and Pérez‐Mellado 1998; Martín and López 2010), in areas where lizard activity is more restricted compared to Castro de São Paio. In contrast, our observations indicate that green lizards, particularly *T. lepidus* , in Castro de São Paio are active almost year‐round. Specifically, we found that *T. lepidus* is more active during the day and tends to initiate its activity earlier as temperature rises, potentially linked to foraging strategies and thermoregulatory behavior (Bergallo and Rocha 1993). It is important to recognize that in some of our multifactorial analyses that included multiple interactions may have been limited by the relatively low number of observations for each factor, limiting statistical power. Although some overall tests showed significant relationships, not all post hoc comparisons did. This pattern is likely to reflect the reduced capacity to detect pairwise differences with small sample sizes. Further studies with larger datasets could help confirm the trends observed and provide more statistical resolution. Our data reveal distinct activity patterns between *T. lepidus* and *L*. *schreiberi*, particularly during spring, likely driven by the breeding period (Carretero 2006; Mateo 2017). During this season, temporal differences are more pronounced, with *T. lepidus* being more active before noon and at midday, while *L. schreiberi's* activity peaks in the afternoon (Figure 5). Although both species reproduce during similar periods (Mateo 2017; Marco 2017), their activity may vary due to differences in microhabitat use and responses to environmental factors such as temperature and humidity. For instance, *T. lepidus* , with its larger body size and greater thermal inertia, likely selects warmer, drier microhabitats like rocky substrates in the morning, enabling faster heat absorption (Goldenberg et al. 2021). In contrast, *L. schreiberi*, being smaller and more sensitive to thermal and humidity fluctuations, may prefer cooler, more humid microhabitats later in the day to avoid overheating and dehydration (Stevenson 1985). While the average body, microhabitat, and environmental temperatures do not differ significantly between species, temporal analyses show that *L. schreiberi* experiences higher body temperatures around midday and lower temperatures in the afternoon (Figure A4). This may reflect physiological adjustments to meet reproductive demands while maintaining water balance. On the other hand, microhabitats with denser vegetation likely provide greater shade and thus require more time to reach thermally optimal conditions for *L. schreiberi*. Consequently, the structural characteristics of each microhabitat may play a critical role in shaping the temporal niche of both species. These findings highlight that temporal niche differences, influenced by seasonal variation in behavior and microhabitat use, could potentially play a critical role in reducing competition and promoting coexistence between these two species.

Finally, our study provides valuable insights into the ecological and phenotypic differences associated with species coexistence, with emphasis on our study system *Timon lepidus* and *Lacerta schreiberi*. Although our study is based on a single population, our approach allowed us to investigate microhabitat use and activity with high spatial and temporal resolution. Extending this work to multiple populations would undoubtedly strengthen the generality of the results obtained and help assess temporal and spatial niche‐related variation in both species. By integrating laboratory experimental findings with real‐world field observations, we found a general congruence between the two, especially in relation to thermal preferences and water balance. However, our field observations also reveal that spatial and temporal factors such as microhabitat use and activity patterns play a crucial and complementary role in shaping niche differentiation. Body size appears as a fundamental trait influencing most traits and is much more evident in laboratory‐derived variables (Reyes‐Puig et al. 2024). These findings underline the importance of combining experimental and field approaches to comprehensively understand niche differences, not only in green lizards but also in other organisms. By highlighting the interplay between physiological and ecological factors, our study contributes to a broader understanding of how coexistence is maintained in ecologically similar species.

## Author Contributions

**Carolina Reyes‐Puig:** conceptualization (lead), data curation (lead), formal analysis (lead), funding acquisition (equal), investigation (lead), methodology (lead), project administration (lead), resources (equal), validation (lead), writing – original draft (lead), writing – review and editing (lead). **Urtzi Enriquez‐Urzelai:** conceptualization (equal), formal analysis (supporting), investigation (supporting), methodology (supporting), supervision (lead), writing – original draft (supporting), writing – review and editing (supporting). **Neftalí Sillero:** formal analysis (supporting), methodology (supporting), supervision (lead), writing – original draft (supporting), writing – review and editing (supporting). **Antigoni Kaliontzopoulou:** conceptualization (equal), formal analysis (supporting), funding acquisition (equal), investigation (supporting), methodology (equal), resources (equal), supervision (lead), writing – original draft (supporting), writing – review and editing (supporting).

## Ethics Statement

The entire process of observing and collecting green lizards was conducted under the collections and research permits LICENÇA N° 344‐348/2022 issued by the Instituto da Conservação da Natureza e Florestas, and all methods were reported to CIBIO's ethical committee (Órgão Responsável pelo Bem‐Estar dos Animais, ORBEA).

## Conflicts of Interest

The authors declare no conflicts of interest.

## Supporting information


Appendix S1

Appendix S2

Appendix S3

## Data Availability

All the information is available in the article and in Appendix S1 (raw data from fieldwork) and in Appendices S2 and S3 (codes used for data analysis).

## Appendix B

**TABLE B1:** Summary of environmental variables and body temperature measurements across species, sexes, months, behaviours, and microhabitat categories.

Factor	Species	Category (sample size)	Air T°C	Relative humidity %	Substrate microhabitat T°C	Illuminance (lx)	Body T°C
Sex	*T. lepidus*	Males (*n* = 83)	22.53 ± 3.6	50.9 ± 12.2	29.4 ± 5.1	61.9 ± 10.9	29.9 ± 2.6
		Females (*n* = 44)	22.29 ± 2.5	50.1 ± 11.8	30.6 ± 6.3	65.3 ± 11	29.6 ± 2.9
	*L. schreiberi*	Males (*n* = 53)	23.23 ± 2.6	58.1 ± 6.2	30.2 ± 4.3	63.4 ± 7.5	29.2 ± 2.3
		Females (*n* = 24)	23.23 ± 2.5	54.1 ± 4.7	30.6 ± 4.7	61.4 ± 9.2	29.2 ± 4.5
Month	*T. lepidus*	January (*n* = 2)	18.1 ± 2.8	52.2 ± 4.9	18.2 ± 3.1	—	—
		February (*n* = 17)	21.2 ± 2.2	40.0 ± 11.3	30.1 ± 5.7	—	—
		March (*n* = 24)	19.2 ± 1.9	40.3 ± 7.1	25.7 ± 4.5	—	—
		April (*n* = 7)	21.2 ± 2.6	75.7 ± 5.4	23.5 ± 1.4	—	—
		May (*n* = 34)	24.3 ± 4.3	49.8 ± 7.6	29.8 ± 5.7	60.5 ± 9.8	29.0 ± 3.4
		June (*n* = 37)	22.6 ± 1.6	59.8 ± 8.8	30.7 ± 4.1	68.0 ± 8.0	29.8 ± 3.0
		July (*n* = 13)	23.3 ± 2.4	53.5 ± 6.1	32.5 ± 5.3	54.6 ± 9.7	29.9 ± 2.6
	*L. schreiberi*	March (*n* = 5)	20.3 ± 3.4	52.2 ± 4.8	26.0 ± 6.8	—	—
		April (*n* = 4)	19.2 ± 0.01	68.3 ± 1.5	29.3 ± 0.7	—	—
		May (*n* = 27)	24.4 ± 2.6	57.5 ± 4.6	30.3 ± 5.1	61.3 ± 7.9	28.9 ± 2.2
		June (*n* = 32)	22.5 ± 1.7	56.5 ± 6.9	31.1 ± 3.8	67.7 ± 6.4	28.5 ± 3.5
		July (*n* = 11)	23.8 ± 2.8	54.9 ± 2.9	30.9 ± 4.4	56.4 ± 6	32.4 ± 3.3
Behaviour	*T. lepidus*	Basking (*n* = 88)	22.3 ± 3.0	50.2 ± 12.5	30.0 ± 5.4	64.1 ± 10.4	29.2 ± 2.8
		Fleeing (*n* = 11)	23.7 ± 4.9	44.8 ± 6.8	26.1 ± 5.1	62.4 ± 6.2	31.5 ± 1.2
		Foraging (*n* = 10)	21.7 ± 1.9	57.9 ± 12.2	30.5 ± 5.4	59.3 ± 9.5	29.6 ± 3.6
		Mating (*n* = 8)	24.9 ± 0.1	58.5 ± 1.2	34.3 ± 0.0001	75.3 ± 0.9	31.6 ± 0.4
		Sheltering (*n* = 17)	21.7 ± 4.6	47.9 ± 12.1	27.8 ± 6.8	46.9 ± 6.5	30.4 ± 2.2
	*L. schreiberi*	Basking (*n* = 53)	23.4 ± 2.7	57.0 ± 6.2	30.9 ± 4.1	63.9 ± 6.8	28.8 ± 2.7
		Fleeing (*n* = 6)	22.5 ± 2.1	58.0 ± 4.7	23.9 ± 1.3	60.2 ± 5.9	26.3 ± 2.1
		Foraging (*n* = 8)	23.1 ± 1.3	58.5 ± 1.7	27.8 ± 2.5	63.0 ± 13.1	32.2 ± 1.3
		Mating (*n* = 8)	20.5 ± 0.2	51 ± 7.0	34.6 ± 3.0	66.2 ± 1.7	31.8 ± 0.2
		Sheltering (*n* = 5)	24.2 ± 2.7	59.8 ± 5.6	28.2 ± 4.7	47.4 ± 7.2	28.6 ± 2.7
Microhabitat	*T. lepidus*	Rocky substrate (*n* = 88)	22.4 ± 3.3	50.9 ± 12.8	29.5 ± 5.1	64.3 ± 11.7	30.0 ± 2.4
		Herbaceous vegetation (*n* = 13)	23.0 ± 2.2	51.5 ± 9.6	28.7 ± 4.2	59.6 ± 8.3	29.7 ± 2.2
		Ground (*n* = 5)	22.6 ± 3.8	46.6 ± 6.2	32.8 ± 9.9	48.4 ± 10.7	29.4 ± 3.0
		Cement/rubble (*n* = 12)	20.8 ± 2.9	49.9 ± 11.3	31.0 ± 6.9	66.3 ± 5.5	28.9 ± 3.9
		Walkway (*n* = 5)	25.1 ± 5.3	50.2 ± 13	34.1 ± 9.0	65.2 ± 7.3	28.2 ± 5.2
	*L. schreiberi*	Rocky substrate (*n* = 5)	24.5 ± 2.8	59.4 ± 0.6	34 ± 6.8	60.8 ± 5.6	30.4 ± 0.3
		Herbaceous vegetation (*n* = 63)	23.1 ± 2.4	56.3 ± 6.0	29.9 ± 4.3	63.5 ± 8.5	29.2 ± 3.5
		Ground (*n* = 5)	26.8 ± 1.3	53.3 ± 1.0	32.2 ± 0.6	64.5 ± 0.6	28.8 ± 0.9
		Cement/rubble (*n* = 5)	19.8 ± 1.2	66.3 ± 4.3	29.4 ± 0.7	54.1 ± 1.3	25.4 ± 2.3

*Note:* The table presents mean values (±standard deviation) for air temperature (°C), relative humidity (%), substrate microhabitat temperature (°C), illuminance (lx), and body temperature (°C) for *T. lepidus* and *L. schreiberi*. Sample sizes (*n*) are indicated for each category.

## Appendix C

**TABLE C2:** Results of ANOVAs performed on several variables recorded in the field to test for differences between the two species (Sp) of green lizards (*Timon lepidus* and *Lacerta shreiberi*).

	df	SS	Rsq	*Z*	Pr(>*F*)
Air temperature ~ Species * Sex					
Sp	1	24.03	0.013	1.180	0.123
Sex	1	0.71	4 × 10^−4^	−0.837	0.786
Sp*Sex	1	0.83	4.7 × 10^−4^	−0.755	0.761
Residuals	186	1759.07	0.985		
Total	189	1784.64			
Relative humidity ~ Species * Sex					
Sp	1	1621.1	0.084	2.925	**0.001****
Sex	1	133.5	0.006	0.501	0.257
Sp*Sex	1	93.7	0.004	0.323	0.359
Residuals	186	19,867.6	0.91		
Total	189	21,715.9			
Illuminance ~ Species * Sex					
Sp	1	188.6	0.015	1.108	0.140
Sex	1	4.5	3.6 × 10^−4^	−1.002	0.824
Sp*Sex	1	58.6	0.004	0.215	0.443
Residuals	140	12,203.1	0.977		
Total	143	12,454.8			
Body temperature ~ Species * Sex					
Sp	1	10.81	0.010	0.569	0.306
Sex	1	0.41	4 × 10^−4^	−1.008	0.829
Sp*Sex	1	0.45	4.5 × 10^−4^	−0.981	0.821
Residuals	111	1005.75	0.988		
Total	114	1017.43			
Microhabitat substrate temperature ~ Species * Sex					
Sp	1	10.9	0.002	−0.068	0.539
Sex	1	35.0	0.007	0.705	0.250
Sp*Sex	186	6.6	0.001	−0.269	0.624
Residuals	189	4683.7	0.988		
Total		4736.2			

Bold *p*‐values indicate statistically significant differences after multiple comparisons.

Abbreviations: df, degrees of freedom; Rsq, *R*‐squared; SS, sums of squares.

Significance levels are denoted as follows: ****p* < 0.001; ***p* < 0.01; *p* < 0.05.

**TABLE C1:** Results of ANOVAs performed on several microhabitat variables recorded in the field to test for differences between the two species (Sp) of green lizards (*Timon lepidus* and *Lacerta shreiberi*) and microhabitat.

	df	SS	Rsq	*Z*	Pr(>*F*)
Air temperature ~ Species * Microhabitat					
Sp	1	35.27	0.057	1.666	0.05
Microhabitat	3	94.71	0.027	2.182	**0.015***
Sp*Microhabitat	3	45.25	0.89	1.082	0.148
Residuals	177	1482.92			
Total	184	1658.15			
Relative humidity ~ Species * Microhabitat					
Sp	1	1686.1	0.080	3.051	**0.001****
Microhabitat	3	239.5	0.011	−0.118	0.538
Sp*Microhabitat	3	316.2	0.015	0.219	0.415
Residuals	177	18,814.0	0.893		
Total	184	21,055.8			
Illuminance ~ Species * Microhabitat					
Sp	1	9.1	0.0006	−0.713	0.758
Microhabitat	3	284.0	0.020	0.254	0.409
Sp*Microhabitat	3	1022.7	0.075	2.161	**0.015***
Residuals	132	12,277.2	0.903		
Total	139	13,593.0			
Body temperature ~ Species * Microhabitat					
Sp	1	11.03	0.011	0.677	0.271
Microhabitat	3	18.93	0.020	−0.103	0.544
Sp*Microhabitat	3	10.74	0.114	−0.566	0.700
Residuals	103	895.83	0.956		
Total	110	936.53			
Microhabitat substrate temperature ~ Species * Microhabitat					
Sp	1	16.2	0.003	0.183	0.440
Microhabitat	3	90.4	0.019	0.516	0.302
Sp*Microhabitat	3	72.9	0.016	0.206	0.423
Residuals	169	4345.6	0.960		
Total	176	4525.1			

Abbreviations: df, degrees of freedom; Rsq, *R*‐squared; SS, sums of squares.

**TABLE C3:** Post hoc pairwise derived from the significant effect of interaction in ANOVA (Illuminance ~ Species * Microhabitat), showing the effect size (*d*), the 95% upper confidence limit (UCL‐95%), the *Z‐*value, and the associated *p*‐value (Pr).

	*d*	UCL (95%)	Z	Pr > d
*Ls*.cement/rubble:*Tl*.cement/rubble	12.175	11.909	1.642	**0.042**
*Ls*.cement/rubble:*Ls*.ground	10.425	16.464	0.788	0.226
*Ls*.cement/rubble:*Tl*.ground	5.708	18.227	−0.170	0.577
*Ls*.cement/rubble:*Ls*.herbaceous vegetation	9.419	9.941	1.425	**0.075**
*Ls*.cement/rubble:*Tl*.herbaceous vegetation	5.534	11.184	0.427	0.372
*Ls*.cement/rubble:*Ls*.rocky substrate	6.745	14.177	0.476	0.348
*Ls*.cement/rubble:*Tl*.rocky substrate	10.255	11.102	1.513	**0.069**
*Tl*.cement/rubble:*Ls*.ground	1.750	14.229	−0.885	0.799
*Tl*.cement/rubble:*Tl*.ground	17.883	16.163	1.761	**0.027**
*Tl*.cement/rubble:*Ls*.herbaceous vegetation	2.756	7.708	0.079	0.483
*Tl*.cement/rubble:*Tl*.herbaceous vegetation	6.641	8.788	1.058	0.163
*Tl*.cement/rubble:*Ls*.rocky substrate	5.430	12.106	0.308	0.414
*Tl*.cement/rubble:*Tl*.rocky substrate	1.920	8.423	−0.402	0.652
*Ls*.ground:*Tl*.ground	16.133	14.237	1.741	**0.032**
*Ls*.ground:*Ls*.herbaceous vegetation	1.006	14.115	−1.392	0.909
*Ls*.ground:*Tl*.herbaceous vegetation	4.891	14.287	0.052	0.491
*Ls*.ground:*Ls*.rocky substrate	3.680	17.293	−0.611	0.734
*Ls*.ground:*Tl*.rocky substrate	0.170	14.738	−2.203	0.989
*Tl*.ground:*Ls*.herbaceous vegetation	15.127	16.330	1.492	**0.065**
*Tl*.ground:*Tl*.herbaceous vegetation	11.242	15.691	0.999	0.170
*Tl*.ground:*Ls*.rocky substrate	12.453	19.607	0.750	0.249
*Tl*.ground:*Tl*.rocky substrate	15.964	16.556	1.453	**0.068**
*Ls*.herbaceous vegetation:*Tl*.herbaceous vegetation	3.885	6.459	0.710	0.248
*Ls*.herbaceous vegetation:*Ls*.rocky substrate	2.674	9.390	−0.225	0.580
*Ls*.herbaceous vegetation:*Tl*.rocky substrate	0.836	4.216	−0.557	0.714
*Tl*.herbaceous vegetation:*Ls*.rocky substrate	1.211	11.415	−1.059	0.835
*Tl*.herbaceous vegetation:*Tl*.rocky substrate	4.721	7.364	0.834	0.209
*Ls*.rocky substrate:*Tl*.rocky substrate	3.510	9.500	0.120	0.473

Bold *p*‐values indicate statistically significant differences after multiple comparisons.

Significance levels are denoted as follows: ****p* < 0.001; ***p* < 0.01; *p* < 0.05.

**TABLE C4:** Post hoc pairwise derived from the significant effect of interaction in ANOVA (Body temperature ~ Species * Month), showing the effect size (*d*), the 95% upper confidence limit (UCL‐95%), the Z‐value, and the associated *p*‐value (Pr).

	*d*	UCL (95%)	Z	Pr
*Ls*.May:*Tl*.May	0.877	1.946	0.302	0.402
*Ls*.May:*Ls*.June	0.399	1.656	−0.391	0.652
*Ls*.May:*Tl*.June	0.894	1.743	0.522	0.318
*Ls*.May:*Ls*.July	3.558	3.765	1.479	**0.067**
*Ls*.May:*Tl*.July	1.109	4.079	−0.970	0.819
*Tl*.May:*Ls*.June	1.276	2.029	0.713	0.260
*Tl*.May:*Tl*.June	0.016	1.661	−2.118	0.986
*Tl*.May:*Ls*.July	2.681	3.043	1.301	0.107
*Tl*.May:*Tl*.July	0.232	3.357	−1.725	0.947
*Ls*.June:*Tl*.June	1.292	1.902	0.933	0.199
*Ls*.June:*Ls*.July	3.957	3.740	1.661	**0.038***
*Ls*.June:*Tl*.July	1.508	3.988	−0.788	0.789
*Tl*.June:*Ls*.July	2.664	3.253	1.189	0.116
*Tl*.June:*Tl*.July	0.215	3.419	−1.929	0.971
*Ls*.July:*Tl*.July	2.449	2.773	1.391	**0.084**

Abbreviations: *Ls*, *Lacerta schreiberi*; *Tl*, *Timon lepidus*.

**TABLE C5:** Results of ANOVAs performed on several variables recorded in the field to test for differences between the two species (Sp) of green lizards (*Timon lepidus* and *Lacerta shreiberi*) and behaviour.

	df	SS	Rsq	*Z*	Pr(>*F*)
Air temperature ~ Species * Behaviour					
Sp	1	27.58	0.015	1.395	**0.07**.
Behaviour	4	18.13	0.010	−0.544	0.719
Sp*Behaviour	4	97.61	0.054	1.786	**0.033***
Residuals	180	1655.59	0.920		
Total	189	1799.01			
Relative humidity ~ Species * Behaviour					
Sp	1	1721.2	0.079	3.088	**0.001****
Behaviour	4	754.6	0.034	1.120	0.139
Sp*Behaviour	4	834.7	0.038	1.288	0.105
Residuals	180	18,462.1	0.847		
Total	189	21,772.6			
Illuminance ~ Species * Behaviour					
Sp	1	13.0	0.0009	−0.357	0.649
Behaviour	4	3458.6	0.279	5.322	**0.001****
Sp*Behaviour	4	257.9	0.018	0.039	0.482
Residuals	134	9742.9	0.707		
Total	143	13,772.3			
Body temperature ~ Species * Behaviour					
Sp	1	10.01	0.009	0.681	0.265
Behaviour	4	82.20	0.080	1.685	**0.042***
Sp*Behaviour	4	53.68	0.052	0.954	0.164
Residuals	105	872.75	0.856		
Total	114	1018.65			
Microhabitat substrate temperature ~ Species * Behaviour					
Sp	1	10.9	0.002	−0.014	0.517
Behaviour	4	548.5	0.115	3.332	**0.001****
Sp*Behaviour	4	54.1	0.011	−0.530	0.705
Residuals	180	4122.7	0.870		
Total	189	4736.2			

Abbreviations: df, degrees of freedom; Rsq, *R*‐squared; SS, sums of squares.

**TABLE C6:** Post hoc pairwise derived from the significant effect of interaction in ANOVA (Air temperature ~ Species * Behaviour), showing the effect size (*d*), the 95% upper confidence limit (UCL‐95%), the *Z*‐value, and the associated *p*‐value (Pr).

	*d*	UCL (95%)	*Z*	Pr > *d*
*Ls*.basking:*Tl*.basking	1.186	1.705	0.766	0.241
*Ls*.basking:*Ls*.fleeing	0.949	3.172	−0.051	0.534
*Ls*.basking:*Tl*.fleeing	0.229	2.196	−1.024	0.826
*Ls*.basking:*Ls*.foraging	0.399	3.036	−0.943	0.813
*Ls*.basking:*Tl*.foraging	1.727	3.070	0.416	0.376
*Ls*.basking:*Ls*.mating	2.949	3.695	1.286	0.102
*Ls*.basking:*Tl*.mating	1.476	2.238	0.914	0.188
*Ls*.basking:*Ls*.sheltering	0.726	3.193	−0.408	0.651
*Ls*.basking:*Tl*.sheltering	1.728	2.526	0.756	0.249
*Tl*.basking:*Ls*.fleeing	0.237	3.909	−1.422	0.920
*Tl*.basking:*Tl*.fleeing	1.415	2.438	0.718	0.254
*Tl*.basking:*Ls*.foraging	0.787	2.954	−0.225	0.599
*Tl*.basking:*Tl*.foraging	0.541	2.243	−0.465	0.672
*Tl*.basking:*Ls*.mating	1.763	4.407	0.194	0.426
*Tl*.basking:*Tl*.mating	2.662	2.665	1.581	**0.052**
*Tl*.basking:*Ls*.sheltering	1.912	3.322	0.775	0.229
*Tl*.basking:*Tl*.sheltering	0.542	1.837	−0.150	0.580
*Ls*.fleeing:*Tl*.fleeing	1.178	3.791	−0.054	0.542
*Ls*.fleeing:*Ls*.foraging	0.550	4.436	−1.012	0.837
*Ls*.fleeing:*Tl*.foraging	0.778	4.835	−0.879	0.796
*Ls*.fleeing:*Ls*.mating	2.000	4.271	0.450	0.344
*Ls*.fleeing:*Tl*.mating	2.425	3.763	0.890	0.200
*Ls*.fleeing:*Ls*.sheltering	1.675	4.590	0.143	0.470
*Ls*.fleeing:*Tl*.sheltering	0.779	4.425	−0.750	0.761
*Tl*.fleeing:*Ls*.foraging	0.628	3.414	−0.574	0.707
*Tl*.fleeing:*Tl*.foraging	1.956	3.632	0.562	0.314
*Tl*.fleeing:*Ls*.mating	3.178	4.345	1.187	0.121
*Tl*.fleeing:*Tl*.mating	1.247	2.853	0.352	0.381
*Tl*.fleeing:*Ls*.sheltering	0.497	3.536	−0.896	0.796
*Tl*.fleeing:*Tl*.sheltering	1.956	2.990	0.807	0.239
*Ls*.foraging:*Tl*.foraging	1.328	3.863	0.086	0.480
*Ls*.foraging:*Ls*.mating	2.550	4.831	0.576	0.298
*Ls*.foraging:*Tl*.mating	1.875	3.649	0.537	0.315
*Ls*.foraging:*Ls*.sheltering	1.125	4.047	−0.263	0.607
*Ls*.foraging:*Tl*.sheltering	1.329	3.330	0.261	0.403
*Tl*.foraging:*Ls*.mating	1.222	5.340	−0.539	0.720
*Tl*.foraging:*Tl*.mating	3.203	3.686	1.304	**0.093**
*Tl*.foraging:*Ls*.sheltering	2.453	4.248	0.749	0.226
*Tl*.foraging:*Tl*.sheltering	0.001	2.439	−2.641	1.000
*Ls*.mating:*Tl*.mating	4.425	4.003	1.748	**0.031***
*Ls*.mating:*Ls*.sheltering	3.675	4.970	1.127	0.132
*Ls*.mating:*Tl*.sheltering	1.221	4.822	−0.464	0.684
*Tl*.mating:*Ls*.sheltering	0.750	3.658	−0.536	0.703
*Tl*.mating:*Tl*.sheltering	3.204	3.384	1.512	**0.064**
*Ls*.sheltering:*Tl*.sheltering	2.454	3.834	0.929	0.187

Bold *p*‐values indicate statistically significant differences after multiple comparisons.

Abbreviations: *Ls*, *Lacerta schreiberi*; *Tl*, *Timon lepidus*.

Significance levels are denoted as follows: ****p* < 0.001; ***p* < 0.01; *p* < 0.05.

**TABLE C7:** Post hoc pairwise comparisons derived from the significant effect of behaviour in ANOVA (Illuminance ~ Species * Behaviour), showing the effect size (*d*), the 95% upper confidence limit (UCL‐95%), the *Z*‐value, and the associated *p*‐value (Pr).

	*d*	UCL‐0.95	*Z*	Pr
basking: fleeing	2.384	7.411	−0.062	0.542
basking: foraging	3.473	8.002	0.060	0.506
basking: mating	8.261	11.978	−0.029	0.519
basking: sheltering	16.926	21.881	0.040	0.493
fleeing: foraging	1.089	7.229	−0.864	0.777
fleeing: mating	10.644	16.548	−0.001	0.503
fleeing: sheltering	14.542	20.999	0.014	0.499
foraging: mating	11.733	17.164	−0.010	0.498
foraging: sheltering	13.453	19.378	0.030	0.486
mating: sheltering	25.187	31.407	0.029	0.470

Bold *p*‐values indicate statistically significant differences after multiple comparisons.

Significance levels are denoted as follows: ****p* < 0.001; ***p* < 0.01; *p* < 0.05.

**TABLE C8:** Post hoc pairwise comparisons derived from the significant effect of behaviour in ANOVA (Body temperature ~ Species * Behaviour), showing the effect size (*d*), the 95% upper confidence limit (UCL‐95%), the *Z*‐value, and the associated *p*‐value (Pr).

	*d*	UCL‐0.95	*Z*	Pr
basking: fleeing	0.389	2.637	−0.863	0.798
basking: foraging	1.629	3.200	0.021	0.509
basking: mating	2.644	4.100	−0.009	0.501
basking: sheltering	0.555	2.527	−0.501	0.684
fleeing: foraging	1.240	3.921	−0.133	0.551
fleeing: mating	2.255	4.836	0.068	0.508
fleeing: sheltering	0.166	3.300	−1.417	0.901
foraging: mating	1.015	3.061	−0.068	0.556
foraging: sheltering	1.074	3.577	−0.045	0.527
mating: sheltering	2.089	4.375	0.117	0.488

Bold *p*‐values indicate statistically significant differences after multiple comparisons.

Significance levels are denoted as follows: ****p* < 0.001; ***p* < 0.01; *p* < 0.05.

**TABLE C9:** Post hoc pairwise derived from the significant effect of behaviour in ANOVA (microhabitat substrate temperature ~ Species * Behaviour), showing the effect size (*d*), the 95% upper confidence limit (UCL‐95%), the *Z*‐value, and the associated *p*‐value (Pr).

	d	UCL‐0.95	*Z*	Pr
basking: fleeing	5.096	7.687	0.081	0.459
basking: foraging	0.642	3.176	−0.506	0.680
basking: mating	4.057	6.449	0.025	0.482
basking: sheltering	2.424	4.315	0.042	0.516
fleeing: foraging	4.453	8.047	0.093	0.462
fleeing: mating	9.153	12.616	0.077	0.479
fleeing: sheltering	2.672	5.803	0.137	0.476
foraging: mating	4.699	8.008	0.043	0.496
foraging: sheltering	1.781	4.960	0.014	0.516
mating: sheltering	6.481	9.387	−0.005	0.500

Bold *p*‐values indicate statistically significant differences after multiple comparisons.

Significance levels are denoted as follows: ****p* < 0.001; ***p* < 0.01; *p* < 0.05.

**TABLE C10:** Results of ANOVAs performed on several variables recorded in the field to test for differences between the two species (Sp) of green lizards (*Timon lepidus* and *Lacerta shreiberi*) between months.

	df	SS	Rsq	*Z*	Pr(>*F*)
Air temperature ~ Species * Month					
Sp	1	0.48	0.0004	−0.962	0.817
Month	2	95.79	0.085	2.692	**0.004****
Sp*Month	2	3.64	0.003	−0.798	0.767
Residuals	135	1020.62	0.910		
Total	140	1120.53			
Relative humidity ~ Species * Month					
Sp	1	381.8	0.046	2.194	**0.001****
Month	2	490.5	0.060	2.431	**0.005****
Sp*Month	2	613.4	0.075	2.765	**0.003****
Residuals	135	6671.6	0.817	0.817	
Total	140	8157.2			
Illuminance ~ Species * Month					
Sp	1	0.4	4 × 10^−5^	−1.615	0.941
Month	2	3298.4	0.269	5.347	**0.001****
Sp*Month	2	60.2	0.004	−0.409	0.668
Residuals	129	8897.3	0.725		
Total	134	12,256.3			
Body temperature ~ Species * Month					
Sp	1	10.81	0.010	0.633	0.283
Month	2	50.03	0.050	1.613	**0.047***
Sp*Month	2	50.61	0.050	1.590	**0.052**.
Residuals	109	905.97	0.890		
Total	114	1017.43			
Microhabitat substrate temperature ~ Species * Month					
Sp	1	28.97	0.009	0.675	0.274
Month	2	68.43	0.228	0.765	0.219
Sp*Month	2	18.94	0.006	−0.421	0.681
Residuals	125	2881.86	0.961		
Total	130	2998.21			

Bold *p*‐values indicate statistically significant differences after multiple comparisons.

Abbreviations: df, degrees of freedom; Rsq, *R*‐squared; SS, sums of squares.

Significance levels are denoted as follows: ****p* < 0.001; ***p* < 0.01; *p* < 0.05.

**TABLE C11:** Post hoc pairwise derived from the significant effect of months in ANOVA (Air temperature ~ Species * Month), showing the effect size (*d*), the 95% upper confidence limit (UCL‐95%), the *Z*‐value, and the associated *p*‐value (Pr).

	*d*	UCL‐95%	*Z*	Pr
May:June	0.166	1.192	−0.827	0.776
May:July	1.701	3.031	0.070	0.481
June:July	1.867	3.107	0.041	0.494

Bold *p*‐values indicate statistically significant differences after multiple comparisons.

Significance levels are denoted as follows: ****p* < 0.001; ***p* < 0.01; *p* < 0.05.

**TABLE C12:** Post hoc pairwise derived from the significant effect of interaction in ANOVA (Relative humidity ~ Species * Month), showing the effect size (*d*), the 95% upper confidence limit (UCL‐95%), the *Z*‐value, and the associated *p*‐value (Pr).

	d	UCL (95%)	*Z*	Pr > *d*
*Ls*.May:*Tl*.May	8.442	6.656	2.348	**0.005*****
*Ls*.May:*Ls*.June	1.000	7.187	−1.695	0.942
*Ls*.May:*Tl*.June	0.483	3.697	−0.841	0.783
*Ls*.May:*Ls*.July	2.650	5.774	0.334	0.391
*Ls*.May:*Tl*.July	4.000	5.994	0.781	0.230
*Tl*.May:*Ls*.June	7.442	10.552	−0.006	0.490
*Tl*.May:*Tl*.June	7.959	6.822	2.199	**0.013****
*Tl*.May:*Ls*.July	5.792	9.248	0.425	0.367
*Tl*.May:*Tl*.July	4.442	5.181	1.268	0.114
*Ls*.June:*Tl*.June	0.517	6.388	−1.896	0.965
*Ls*.June:*Ls*.July	1.650	7.100	−0.554	0.700
*Ls*.June:*Tl*.July	3.000	10.233	−1.296	0.901
*Tl*.June:*Ls*.July	2.167	5.149	0.178	0.446
*Tl*.June:*Tl*.July	3.517	6.597	0.413	0.373
*Ls*.July:*Tl*.July	1.350	8.517	−0.991	0.814

Bold *p*‐values indicate statistically significant differences after multiple comparisons.

Abbreviations: *Ls*, *Lacerta schreiberi*; *Tl*, *Timon lepidus*.

Significance levels are denoted as follows: ****p* < 0.001; ***p* < 0.01; *p* < 0.05.

**TABLE C13:** Post hoc pairwise derived from the significant effect of months in ANOVA (Illuminance ~ Species * Month), showing the effect size (*d*), the 95% upper confidence limit (UCL‐95%), the *Z*‐value, and the associated *p*‐value (Pr).

	*d*	UCL‐95%	*Z*	Pr
May:June	7.641	10.055	−0.041	0.518
May:July	5.390	8.766	−0.001	0.52
June:July	13.031	16.588	−0.029	0.514

Bold *p*‐values indicate statistically significant differences after multiple comparisons.

Significance levels are denoted as follows: ****p* < 0.001; ***p* < 0.01; *p* < 0.05.

## Appendix D

**TABLE D1:** Model comparison and parameter estimates for several circular models to test differences in time related to body temperature (Tb), environmental temperature (AT), snout‐vent length (SVL), environmental humidity (EH), amount of solar light, and species (*L. schreiberi* and *T. lepidus* ).

Model	DIC	WAIC	R‐hat	Source	Estimate	SD	LB	UB
Time ~ SVL * AT * Species	166.885	169.21	0.944	Intercept	1.731	0.57	1.077	2.789
				Kappa	3.938	0.58	**2.973**	**5.229**
				SVL	−0.112	0.555	−1.122	1.138
				AT	0.539	0.369	−0.076	1.34
				SVL:AT	0.049	0.599	−1.22	1.142
				SVL:Species *T. lepidus*	0.115	0.622	−1.237	1.238
				AT:Species *T. lepidus*	−1.404	0.642	**−2.748**	**−0.235**
				SVL:AT:Species *T. lepidus*	−0.406	0.814	−2.035	1.143
				Species *T. lepidus*	−2.77	0.943	**−4.953**	**−1.487**
Time ~ SVL * AT * EH * Species	165.519	169.569	0.97	Intercept	1.357	0.626	**0.777**	**2.137**
				Kappa	4.059	0.607	**2.966**	**5.333**
				SVL	0.038	0.75	−1.417	1.482
				AT	−0.005	0.645	−1.149	1.381
				EH	−0.744	0.659	−1.984	0.662
				SVL:AT	−0.12	0.753	−1.682	1.334
				SVL:EH	−0.395	0.79	−1.904	1.162
				AT:EH	1.01	0.658	−0.314	2.282
				SVL:Species *T. lepidus*	0.032	0.84	−1.777	1.514
				AT:Species *T. lepidus*	−1.114	0.814	−2.926	0.263
				EH:Species *T. lepidus*	−0.42	0.778	−2.03	1.101
				SVL:AT:EH	0.26	0.755	−1.277	1.729
				SVL:AT:Species *T. lepidus*	−0.493	0.843	−2.264	1.136
				SVL:EH:Species *T. lepidus*	0.223	0.847	−1.624	1.797
				AT:EH:Species *T. lepidus*	−0.536	0.852	−2.301	1.011
				SVL:AT:EH:Species *T. lepidus*	−0.297	0.831	−2.035	1.353
				Species *T. lepidus*	−2.032	0.753	**−3.264**	**−0.325**
Time ~ SVL * Tb * Species	175.28	177.791	0.948	Intercept	−0.486	0.565	−1.697	0.132
				Kappa	3.676	0.533	**2.734**	**4.813**
				SVL	−0.163	0.624	−1.4	1.091
				Eye	0.371	0.315	−0.2	1.043
				SVL:Tb	−0.514	0.66	−1.808	0.834
				SVL:Species *T. lepidus*	1.082	0.826	−0.431	2.698
				Tb:Species *T. lepidus*	0.813	0.791	−0.526	2.533
				SVL: Tb:Species *T. lepidus*	0.912	0.831	−0.582	2.601
				Species *T. lepidus*	1.406	2.015	−5.876	1.387
Time ~ SVL * EH * Species	177.504	179.45	0.936	Intercept	−0.621	0.475	−1.855	0.009
				Kappa	3.683	0.53	**2.685**	**4.74**
				EH	−0.012	0.398	−0.83	0.773
				SVL	−0.163	0.597	−1.278	1.046
				EH:SVL	−0.629	0.66	−2.014	0.469
				EH:Species *T. lepidus*	0.563	0.725	−0.619	2.247
				SVL:Species *T. lepidus*	1.546	0.72	0.161	3.032
				EH:SVL:Species *T. lepidus*	0.389	0.842	−1.105	2.254
				Species *T. lepidus*	1.661	0.943	**−5.738**	**−2.045**
Time ~ SVL * Tb * EH * Species	177.398	178.928	0.974	Intercept	−0.043	0.226	−0.552	0.297
				Kappa	3.662	0.531	**2.726**	**4.794**
				SVL	−0.097	0.839	−1.599	1.61
				Tb	0.361	0.602	−0.754	1.625
				EH	−0.305	0.739	−1.84	1.069
				SVL: Tb	0.063	0.858	−1.544	1.851
				SVL:EH	−0.415	0.809	−2.076	1.092
				Tb:EH	0.055	0.75	−1.454	1.538
				SVL:Species *T. lepidus*	1.08	0.888	−0.678	2.858
				Tb:Species *T. lepidus*	0.901	0.917	−0.863	2.732
				EH:Species *T. lepidus*	0.42	0.896	−1.225	2.199
				SVL:Tb:EH	−0.393	0.814	−1.96	1.3
				SVL:Tb:Species *T. lepidus*	0.829	0.983	−1.14	2.728
				SVL:EH:Species *T. lepidus*	0.52	0.945	−1.334	2.307
				Eye:EH:Species *T. lepidus*	0.481	0.889	−1.224	2.322
				SVL:Tb:EH:Species *T. lepidus*	0.601	0.95	−1.251	2.413
				Species*T. Lepidus*	0.635	1.044	−4.478	1.571
Time ~ SVL * IL * Species	178.478	181.17	0.952	Intercept	−0.513	0.498	−1.987	0.093
				Kappa	3.586	0.521	**2.648**	**4.679**
				SVL	−0.274	0.568	−1.411	0.899
				IL	0.001	0.378	−0.864	0.697
				SVL:IL	−0.609	0.654	−1.914	0.734
				SVL:Species *T. lepidus*	1.395	0.695	**0.194**	**2.889**
				IL:Species *T. lepidus*	0.711	0.759	−0.529	2.381
				SVL:IL:Species *T. lepidus*	0.805	0.771	−0.591	2.384
				Species *T. lepidus*	1.406	2.41	−5.815	1.818

Bold values indicate 95% credible intervals that do not include zero, suggesting statistically meaningful effects..

*Note:* The models' performance is evaluated using Deviance Information Criterion (DIC), Watanabe‐Akaike Information Criterion (WAIC), and R‐hat values; convergence is indicated by values closer to 1 as good convergence. The parameter estimates include the mean (Estimate), standard deviation (SD), and 95% credible intervals (LB, UB).

Abbreviations: AT, environmental temperature; EH, relative humidity; IL, illuminance; SVL, snout to vent length.
