# Supplementary material for: Niche Differences in Coexisting Species: Ecological Insights Into the Role of Activity Patterns, Space Use, and Environmental Preferences
Source: Ecol Evol. 2025 Jul 31;15(8):e71802. doi: 10.1002/ece3.71802 (PMC12314190; doi:10.1002/ece3.71802)
Supplement: Supplementary file 2 — Appendix S2 [file ECE3-15-e71802-s002.docx]

# ---------------------------------------------

# R Script for Environmental, Behavioural and Microhabitat Analysis

# Data source: CastroDBEE - Data of lizards

# Author: Carolina Reyes-Puig (2025)

# Description:

# This script loads, processes, and analyses field data

# to explore species-level differences in body temperature,

# environmental variables (air temp, humidity, light),

# and microhabitat use in two species of green lizards

# in Northern Portugal. It includes visualizations and

# RRPP-based statistical tests.

# ---------------------------------------------

##

library(RRPP) # For randomization-based statistical models

library(ggplot2) # For plotting

library(tidyverse) # For data manipulation and piping

library(ggpubr) # For combining plots

library(ggridges) # For density ridgeline plots

library(gridExtra) # For arranging multiple plots

library(sjPlot) # For summary and visualisation of models

library(sjmisc) # Data manipulation utilities

library(sjlabelled) # For working with labelled data

library(lme4) # Linear mixed-effects models

library(lmerTest) # P-values for mixed models

library(mgcv) # Generalized additive models (GAMs)

CastroDB <- read.csv("CatrosDBEE.csv", sep = ";")

###Format the time in db.T

CastroDB$Time <- format(as.POSIXlt(CastroDB$Time, format = "%H:%M"), format = "%H:%M")

CastroDB$Date <- format(as.Date(CastroDB$Date), format = "%Y-%m-%d")

A.DB <- CastroDB %>% filter(Status == "Adult") ## Filter the info just for adults

str(A.DB)

# Body and environmental temperatures

# Relative humidity

# Light levels

# Stratified by species, sex, microhabitat, and behaviour

### air temperature

tapply(A.DB$Eye, A.DB$Species, summary, na.rm = TRUE)

tapply(A.DB$EnT, A.DB$Species, mean, na.rm = TRUE) #Air temperature

tapply(A.DB$Eye, A.DB$Species, mean, na.rm = TRUE) #body temperature

tapply(A.DB$Eye, A.DB$Species, summary, na.rm = TRUE)

with(A.DB, tapply(A.DB$Eye, list(Species, Sex), mean, na.rm = TRUE ))

with(A.DB, tapply(A.DB$EH, list(Species, Sex), mean, na.rm = TRUE ))

with(A.DB, tapply(A.DB$ST, list(Species, Microhabitat), mean, na.rm = TRUE ))

with(A.DB, tapply(A.DB$EnT, list(Species, Microhabitat), function(x) length(na.omit(x))))

tapply(A.DB$Eye, A.DB$Behaviour, summary, na.rm = TRUE)

#### relative humidity

tapply(A.DB$EH, A.DB$Species, mean, na.rm = TRUE)

with(A.DB, tapply(A.DB$EH, list(Species, Sex), mean, na.rm = TRUE ))

with(A.DB, tapply(A.DB$EH, list(Species, Status), sd, na.rm = TRUE ))

### solar light

tapply(A.DB$Fluxometer, A.DB$Species, sd, na.rm = TRUE)

with(A.DB, tapply(A.DB$Fluxometer, list(Species, Sex), mean, na.rm = TRUE ))

with(A.DB, tapply(A.DB$Fluxometer, list(Species, Status), sd, na.rm = TRUE ))

# Standardise date formats and order categorical variables (e.g., month)

### Ordering months and time formats

ord.month <- c("January", "February", "March", "April", "May", "June", "July")

A.DB$Month <- factor(A.DB$Month, levels = ord.month)

A.DB$Date <- trimws(A.DB$Date)

A.DB$Date <- gsub("[^0-9-]", "", A.DB$Date)

A.DB$Date <- as.Date(A.DB$Date, format = "%Y-%m-%d")

str(A.DB$Date)

head(A.DB$Date)

A.DB$Month <- factor(A.DB$Month, levels = month.name)

# Generate histograms, density plots, and bar plots by species and month

# Create summary plots for temperature, humidity, and light

# Boxplots for body temperature, air temp, RH, and light across time

# Detection and months

Date.act <- ggplot(A.DB, aes(x = Month)) +

geom_bar(aes(color = Species, fill = Species),

alpha = 0.35, position = "identity", show.legend = F) +

scale_fill_manual(values = c("#90EE90", "#00008B")) +

scale_color_manual(values = c("#90EE90", "#00008B")) +

aes(y=stat(count)/sum(stat(count))) +

scale_y_continuous(labels = scales::percent) +

theme_classic() + theme(strip.text.x = element_blank(), axis.title.y = element_text(size =15),

title = element_text(size = 16), axis.text.y = element_text(size = 15),

axis.title = element_text(size = 16, face = "bold"),

axis.text.x = element_text(size = 15, angle = 45, hjust = 1), legend.text = element_text(face = "italic")) +

ylab("Percentage") +

xlab("Month") +

facet_wrap(~ Species) +

ggtitle("A")

### Tb and relative himidity frequencies by species

hit.gt <- ggplot(A.DB, aes(x = Eye)) +

geom_histogram(aes(color = Species, fill = Species), binwidth = 0.9,bins = 30,

alpha = 0.4, position = "identity", show.legend = F) +

scale_fill_manual(values = c("#90EE90", "#00008B")) +

scale_color_manual(values = c("#90EE90", "#00008B")) +

facet_wrap( ~ Species) +

aes(y=stat(count)/sum(stat(count))) +

scale_y_continuous(labels = scales::percent) +

theme_classic() + theme(strip.text.x = element_blank(), axis.title.y = element_text(size =13),

title = element_text(size = 16), axis.text.y = element_text(size = 13),

axis.text.x = element_text(size = 14),

axis.text = element_text(size = 14),

axis.title = element_text(size = 16, face = "bold")) +

ylab("Percentage") +

xlab("Field body temperature °C") +

stat_central_tendency(type = "mean", color = "red", linetype = 2, size = 1) +

stat_central_tendency(type = "median", color = "black", linetype = 2, size = 1) +

ggtitle("B")

hit.gh <- ggplot(A.DB, aes(x = EH)) +

geom_histogram(aes(color = Species, fill = Species), binwidth = 2,bins = 30,

alpha = 0.4, position = "identity", show.legend = F) +

scale_fill_manual(values = c("#90EE90", "#00008B")) +

scale_color_manual(values = c("#90EE90", "#00008B")) +

facet_wrap( ~ Species) +

aes(y=stat(count)/sum(stat(count))) +

scale_y_continuous(labels = scales::percent) +

theme_classic() + theme(strip.text.x = element_blank(), axis.title.y = element_text(size =13),

title = element_text(size = 16), axis.text.y = element_text(size = 13),

axis.text.x = element_text(size = 12),

axis.text = element_text(size = 14),

axis.title = element_text(size = 16, face = "bold"),) +

ylab("Percentage") +

xlab("Relative humidity %") +

stat_central_tendency(type = "mean", color = "red", linetype = 2, size = 1) +

stat_central_tendency(type = "median", color = "black", linetype = 2, size = 1) +

scale_x_continuous(breaks = seq(min(na.omit(A.DB$EH)), max(na.omit(A.DB$EH)), by = 5)) +

ggtitle("C")

### 2 axis plot of environmental temperature and humidity

ord.month <- c("January", "February", "March", "April", "May", "June", "July")

A.DB$Month <- factor(A.DB$Month, levels = ord.month)

A.DB$Date <- trimws(A.DB$Date)

A.DB$Date <- gsub("[^0-9-]", "", A.DB$Date)

A.DB$Date <- as.Date(A.DB$Date, format = "%Y-%m-%d")

str(A.DB$Date)

head(A.DB$Date)

##combined graphic with T and H

summarized_data <- A.DB %>%

group_by(Date, Species) %>%

summarize(EnT_sum = mean(EnT),

EH_sum = mean(EH))

Sys.setlocale("LC_TIME", "C")

Tgen <- ggplot(summarized_data, aes(x = Date)) +

geom_line(aes(y = EnT_sum, colour = Species, group = Species), linetype = "dashed", size = 1, show.legend = F) +

scale_colour_manual(values = c("#90EE90", "#00008B")) +

scale_y_continuous(

name = "Air temperature °C",

limits = c(15, 34),

) +

scale_x_date(

date_labels = "%B", # months names in English

date_breaks = "1 month"

) +

xlab("Month") +

ylab("Air temperature °C") +

theme_minimal() +

theme(

axis.text.x = element_text(angle = 45, hjust = 1, size = 15),

axis.text.y = element_text(size = 14),

legend.text = element_text(size = 14),

legend.title = element_text(size = 14),

axis.title = element_text(size = 16, face = "bold"),

plot.title = element_text(size = 17)

) +

ggtitle("A")

Hgen <- ggplot(summarized_data, aes(x = Date)) +

geom_line(aes(y = EnT_sum, colour = Species, group = Species), linetype = "dashed", size = 1, show.legend = F) +

geom_line(aes(y = EH_sum, colour = Species, group = Species), size = 1, show.legend = F) +

scale_colour_manual(values = c("#90EE90", "#00008B")) +

scale_y_continuous(

name = "Relative humidity %",

limits = c(30, 80),

) +

scale_x_date(

date_labels = "%B", # months names in English

date_breaks = "1 month"

) +

xlab("Month") +

ylab("Relative humidity %") +

theme_minimal() +

theme(

axis.text.x = element_text(angle = 45, hjust = 1, size = 15),

axis.text.y = element_text(size = 14),

legend.text = element_text(size = 14),

legend.title = element_text(size = 14),

axis.title = element_text(size = 16, face = "bold"),

plot.title = element_text(size = 17)

) +

ggtitle("B")

grid.arrange(Tgen, Hgen, hit.gt, hit.gh , ncol = 2) #PDF 9*14

# Linear models using lm.rrpp to test for effects of species, sex, month,

# behaviour, and microhabitat on response variables

# Environmental variables and body temperatures

A.DB$Species <- as.factor(A.DB$Species)

A.DB$Sex <- as.factor(A.DB$Sex)

# air temperature (general)

fit.et <- lm.rrpp(A.DB$EnT ~ A.DB$Species * A.DB$Sex, na.rm = T)

anova(fit.et)

# Environmental humidity

fit.eh <- lm.rrpp(A.DB$EH ~ A.DB$Species * A.DB$Sex, na.rm = T)

anova(fit.eh)

# Amount of light

A.DB_filtered.l <- A.DB[complete.cases(A.DB[c("Fluxometer", "Species", "Sex", "Month", "Microhabitat", "Behaviour")]), ]

fit.l <- lm.rrpp(Fluxometer ~ Species * Sex, data = A.DB_filtered.l)

anova(fit.l)

# Substrate temperature

A.DB_filtered.st <- A.DB[complete.cases(A.DB[c("ST", "Species", "Sex", "Month", "Microhabitat", "Behaviour")]), ]

fit.st <- lm.rrpp(ST ~ Species * Sex, data = A.DB_filtered.st)

anova(fit.st)

# Body temperature

A.DB_filtered.eye <- A.DB[complete.cases(A.DB[c("Eye", "Species", "Sex", "Month", "Microhabitat", "Behaviour")]), ]

fit.bt.s <- lm.rrpp(Eye ~ Species * Sex, data = A.DB_filtered.eye)

anova(fit.bt.s)

fit.bt <- lm.rrpp(Eye ~ Species * Month, data = A.DB_filtered.eye)

anova(fit.bt) # all months but we have differente observations per month

A.DB_filtered.eye <- A.DB_filtered.eye %>%

filter(Month %in% c("May", "June", "July")) # for been the months with complete information for body temperature and majority of observations

A.DB_filtered.eye$Month <- factor(A.DB_filtered.eye$Month, levels = c("May", "June", "July"))

fit.bt <- lm.rrpp(Eye ~ Species * Month, data = A.DB_filtered.eye)

anova(fit.bt)

p1 <- pairwise(fit.bt, groups = A.DB_filtered.eye$Month)

summary(p1, test.type = "dist")

#### air temperature #####

fit.at <- lm.rrpp(A.DB_filtered$EnT ~ A.DB_filtered$Species * A.DB_filtered$Month, na.rm = T)

anova(fit.at)

### substrate temperature ###

fit.st <- lm.rrpp(A.DB_filtered$ST ~ A.DB_filtered$Species * A.DB_filtered$Month, na.rm = T)

anova(fit.st)

box.month <- ggplot(A.DB_filtered.eye, aes(x = Month, y = Eye, fill = Species), show.legend = F) +

geom_boxplot(width = 0.8, lwd = 1, alpha = 0.5, aes(color = Species), show.legend = F) +

theme_bw() +

xlab("Month") +

ylab("Body temperature°C") +

scale_fill_manual(values = c("#90EE90", "#00008B")) +

scale_color_manual(values = c("#90EE90", "#00008B")) + # Use the same colors for the border

theme_classic() +

theme(title = element_text(size = 16), axis.text.x = element_text(),

legend.text = element_text(face = "italic"),

axis.text = element_text(size = 15),

axis.title = element_text(size = 16, face = "bold"),

panel.background = element_blank(),

panel.grid.major = element_line(color = "gray90"),

panel.grid.minor = element_line(color = "gray90"),

axis.line = element_line(color = "black")) +

stat_summary(fun.y="mean", geom="point", size=2, position=position_dodge(width=0.75), color="black", show.legend = F) +

ggtitle("D")

# Relative humidity with months

fit.hm <- lm.rrpp(A.DB_filtered.H$EH ~ A.DB_filtered.H$Species * A.DB_filtered.H$Month, na.rm = T)

anova(fit.hm)

A.DB_filtered.H <- A.DB %>%

filter(Month %in% c("May", "June", "July")) # months with more information for this variable

fit.hm <- lm.rrpp(A.DB_filtered.H$EH ~ A.DB_filtered.H$Species * A.DB_filtered.H$Month, na.rm = T)

anova(fit.hm)

A.DB_filtered.H$Month <- factor(A.DB_filtered.H$Month, levels = month.name)

box.moH <- ggplot(A.DB_filtered.H, aes(x = Month, y = EH, fill = Species), show.legend = F) +

geom_boxplot(width = 0.8, lwd = 1, alpha = 0.5, aes(color = Species), show.legend = F) +

theme_bw() +

xlab("Month") +

ylab("Relative humidity %") +

scale_fill_manual(values = c("#90EE90", "#00008B")) +

scale_color_manual(values = c("#90EE90", "#00008B")) + # Use the same colors for the border

theme_classic() +

theme(title = element_text(size = 16), axis.text.x = element_text(),

legend.text = element_text(face = "italic"),

axis.text = element_text(size = 15),

axis.title = element_text(size = 16, face = "bold"),

panel.background = element_blank(),

panel.grid.major = element_line(color = "gray90"),

panel.grid.minor = element_line(color = "gray90"),

axis.line = element_line(color = "black")) +

stat_summary(fun.y="mean", geom="point", size=2, position=position_dodge(width=0.75), color="black", show.legend = F) +

ggtitle("B")

###### air temperature

box.moaT <- ggplot(A.DB_filtered.H, aes(x = Month, y = EnT, fill = Species), show.legend = F) +

geom_boxplot(width = 0.8, lwd = 1, alpha = 0.5, aes(color = Species), show.legend = F) +

theme_bw() +

xlab("Month") +

ylab("Air temperature °C") +

scale_fill_manual(values = c("#90EE90", "#00008B")) +

scale_color_manual(values = c("#90EE90", "#00008B")) + # Use the same colors for the border

theme_classic() +

theme(title = element_text(size = 16), axis.text.x = element_text(),

legend.text = element_text(face = "italic"),

axis.text = element_text(size = 15),

axis.title = element_text(size = 16, face = "bold"),

panel.background = element_blank(),

panel.grid.major = element_line(color = "gray90"),

panel.grid.minor = element_line(color = "gray90"),

axis.line = element_line(color = "black")) +

stat_summary(fun.y="mean", geom="point", size=2, position=position_dodge(width=0.75), color="black", show.legend = F) +

ggtitle("A")

## Amount of ligth

A.DB_filtered.F <- A.DB %>%

filter(Month %in% c("May", "June", "July")) # months with more information for this variable

fit.al <- lm.rrpp(A.DB_filtered.F$Fluxometer ~ A.DB_filtered.F$Species * A.DB_filtered.H$Month, na.rm = T)

anova(fit.al)

A.DB_filtered.F$Month <- factor(A.DB_filtered.F$Month, levels = month.name)

box.moL <- ggplot(A.DB_filtered.F, aes(x = Month, y = Fluxometer, fill = Species), show.legend = F) +

geom_boxplot(width = 0.8, lwd = 1, alpha = 0.5, aes(color = Species), show.legend = F) +

theme_bw() +

xlab("Month") +

ylab("Illuminance (lx)") +

scale_fill_manual(values = c("#90EE90", "#00008B")) +

scale_color_manual(values = c("#90EE90", "#00008B")) + # Use the same colors for the border

theme_classic() +

theme(title = element_text(size = 16), axis.text.x = element_text(),

legend.text = element_text(face = "italic"),

axis.text = element_text(size = 15),

axis.title = element_text(size = 16, face = "bold"),

panel.background = element_blank(),

panel.grid.major = element_line(color = "gray90"),

panel.grid.minor = element_line(color = "gray90"),

axis.line = element_line(color = "black")) +

stat_summary(fun.y="mean", geom="point", size=2, position=position_dodge(width=0.75), color="black", show.legend = F) +

ggtitle("C")

###############################################################################################

###Variables and behaviour

anova(lm.rrpp(A.DB$EnT ~ A.DB$Species * A.DB$Behaviour, rm.na = T))

anova(lm.rrpp(A.DB$Eye ~ A.DB$Species * A.DB$Behaviour, rm.na = T))

anova(lm.rrpp(A.DB$EH ~ A.DB$Species * A.DB$Behaviour, rm.na = T))

anova(lm.rrpp(A.DB$Fluxometer ~ A.DB$Species * A.DB$Behaviour, rm.na = T))

anova(lm.rrpp(A.DB$ST ~ A.DB$Species * A.DB$Behaviour, rm.na = T))

beh.at <- ggplot(A.DB, aes(x = Behaviour, y = EnT, fill = Species), show.legend = F) +

geom_boxplot(width = 0.8, lwd = 1, alpha = 0.5, aes(color = Species), show.legend = F) +

theme_bw() +

xlab("Behaviour") +

ylab("Air temperature °C") +

scale_fill_manual(values = c("#90EE90", "#00008B")) +

scale_color_manual(values = c("#90EE90", "#00008B")) + # Use the same colors for the border

theme(axis.text.x = element_text(),

legend.text = element_text(face = "italic"),

axis.text = element_text(size = 14),

axis.title = element_text(size = 16, face = "bold"),

panel.background = element_blank(),

panel.grid.major = element_line(color = "gray90"),

panel.grid.minor = element_line(color = "gray90"),

axis.line = element_line(color = "black"), title = element_text(size = 16)) +

stat_summary(fun.y="mean", geom="point", size=2,

position=position_dodge(width=0.75), color="black", show.legend = F) +

ggtitle("B")

beh.box <- ggplot(A.DB, aes(x = Behaviour, y = Eye, fill = Species), show.legend = F) +

geom_boxplot(width = 0.8, lwd = 1, alpha = 0.5, aes(color = Species), show.legend = F) +

theme_bw() +

xlab("Behaviour") +

ylab("Body temperature °C") +

scale_fill_manual(values = c("#90EE90", "#00008B")) +

scale_color_manual(values = c("#90EE90", "#00008B")) + # Use the same colors for the border

theme(axis.text.x = element_text(),

legend.text = element_text(face = "italic"),

axis.text = element_text(size = 14),

axis.title = element_text(size = 16, face = "bold"),

panel.background = element_blank(),

panel.grid.major = element_line(color = "gray90"),

panel.grid.minor = element_line(color = "gray90"),

axis.line = element_line(color = "black"), title = element_text(size = 16)) +

stat_summary(fun.y="mean", geom="point", size=2,

position=position_dodge(width=0.75), color="black", show.legend = F) +

ggtitle("E")

beh.h <- ggplot(A.DB, aes(x = Behaviour, y = EH, fill = Species), show.legend = F) +

geom_boxplot(width = 0.8, lwd = 1, alpha = 0.5, aes(color = Species), show.legend = F) +

theme_bw() +

xlab("Behaviour") +

ylab("Relative humidity %") +

scale_fill_manual(values = c("#90EE90", "#00008B")) +

scale_color_manual(values = c("#90EE90", "#00008B")) + # Use the same colors for the border

theme(axis.text.x = element_text(),

legend.text = element_text(face = "italic"),

axis.text = element_text(size = 14),

axis.title = element_text(size = 16, face = "bold"),

panel.background = element_blank(),

panel.grid.major = element_line(color = "gray90"),

panel.grid.minor = element_line(color = "gray90"),

axis.line = element_line(color = "black"), title = element_text(size = 16)) +

stat_summary(fun.y="mean", geom="point", size=2,

position=position_dodge(width=0.75), color="black", show.legend = F) +

ggtitle("C")

beh.f <- ggplot(A.DB, aes(x = Behaviour, y = Fluxometer, fill = Species), show.legend = F) +

geom_boxplot(width = 0.8, lwd = 1, alpha = 0.5, aes(color = Species), show.legend = F) +

theme_bw() +

xlab("Behaviour") +

ylab("Illuminance (lx)") +

scale_fill_manual(values = c("#90EE90", "#00008B")) +

scale_color_manual(values = c("#90EE90", "#00008B")) + # Use the same colors for the border

theme(axis.text.x = element_text(),

legend.text = element_text(face = "italic"),

axis.text = element_text(size = 14),

axis.title = element_text(size = 16, face = "bold"),

panel.background = element_blank(),

panel.grid.major = element_line(color = "gray90"),

panel.grid.minor = element_line(color = "gray90"),

axis.line = element_line(color = "black"), title = element_text(size = 16)) +

stat_summary(fun.y="mean", geom="point", size=2,

position=position_dodge(width=0.75), color="black", show.legend = F) +

ggtitle("D")

beh <- ggplot(A.DB, aes(x = Month)) +

geom_bar(aes(color = Behaviour, fill = Behaviour),

alpha = 0.35, position = "dodge", show.legend = T) +

scale_fill_manual(values = c("#DAA520", "#6B8E23","#4682B4", "#8B0000", "#696969")) +

scale_color_manual(values = c("#DAA520", "#6B8E23","#4682B4", "#8B0000", "#696969")) +

aes(y=stat(count)/sum(stat(count))) +

scale_y_continuous(labels = scales::percent) +

theme_classic() + theme(strip.text.x = element_blank(), axis.title.y = element_text(size =15),

title = element_text(size = 16), axis.text.y = element_text(size = 15),

axis.title = element_text(size = 16, face = "bold"),

axis.text.x = element_text(size = 15, angle = 45, hjust = 1), legend.text = element_text(size = 13),

legend.position = c(0.08, 0.6)) +

ylab("Percentage") +

xlab("Month") +

facet_wrap(~ Species) +

ggtitle("A")

###############################################################################################

## Space use and microhabitat preferences

A.DB$Microhabitat <- as.factor(A.DB$Microhabitat)

A.DB$Species <- as.factor(A.DB$Species)

A.DB_filtered.M <- A.DB %>%

filter(Microhabitat %in% c("rocky substrate", "ground", "herbaceous vegetation", "cement/rubble"))

fit.sat <- lm.rrpp(A.DB_filtered.M$EnT ~ A.DB_filtered.M$Species * A.DB_filtered.M$Microhabitat, na.rm = T)

anova(fit.sat)

fit.st <- lm.rrpp(A.DB_filtered.M$ST ~ A.DB_filtered.M$Species * A.DB_filtered.M$Microhabitat, na.rm = T)

anova(fit.st)

fit.bts <- lm.rrpp(A.DB_filtered.M$Eye ~ A.DB_filtered.M$Species * A.DB_filtered.M$Microhabitat, na.rm = T)

anova(fit.bts)

fit.hs <- lm.rrpp(A.DB_filtered.M$EH ~ A.DB_filtered.M$Species * A.DB_filtered.M$Microhabitat, na.rm = T)

anova(fit.hs)

fit.fs <- lm.rrpp(A.DB_filtered.M$Fluxometer ~ A.DB_filtered.M$Species * A.DB_filtered.M$Microhabitat, na.rm = T)

anova(fit.fs)

# Subset data for dominant microhabitats by species

# Run separate models for comparisons within and across microhabitats

# Boxplots visualising body temp, substrate temp, RH, and light by habitat

#Subsetting T. lepidus in rocky subtrate since is where >70% of observations happened

TL.rb <- A.DB_filtered.F %>% filter(Microhabitat == "rocky substrate", Species == "T. lepidus")

#Subsetting L. schreiberi in herbaceous vegetation since is where >70% of observations happened

LS.hv <- A.DB_filtered.F %>% filter(Microhabitat == "herbaceous vegetation", Species == "L. schreiberi")

#Test differences in temperatures but just in the most common microhabitats

# Substrate temperature

fit.TLs <- lm.rrpp(TL.rb$ST ~ TL.rb$Month, na.rm = T)

anova(fit.TLs)

fit.LSs <- lm.rrpp(LS.hv$ST ~ LS.hv$Month, na.rm = T)

anova(fit.LSs)

# Body temperature

fit.TLe <- lm.rrpp(TL.rb$Eye ~ TL.rb$Month, na.rm = T)

anova(fit.TLe)

fit.LSe <- lm.rrpp(LS.hv$Eye ~ LS.hv$Month, na.rm = T)

anova(fit.LSe)

Subs <- rbind(TL.rb,LS.hv)

Subs$Month <- factor(Subs$Month, levels = ord.month)

### Susbtrate temperatures

box.subs <- ggplot(Subs, aes(x = Species, y = ST, fill = Microhabitat)) +

geom_boxplot(aes(color = Month), alpha = 0.9) + # Set color aesthetic here

scale_fill_manual(values = c("#006400", "#545454")) +

scale_color_manual(values = c("#654321", "#FF6666", "darkorange")) + # Use the same colors for the border

theme(axis.text.x = element_text(face = "italic"),

axis.text = element_text(size = 14),

axis.title = element_text(size = 16, face = "bold"),

panel.background = element_blank(),

panel.grid.major = element_line(color = "gray90"),

panel.grid.minor = element_line(color = "gray90"),

axis.line = element_line(color = "black"),

title = element_text(size = 16)) +

stat_summary(fun.y="mean", geom="point", size=2,

position=position_dodge(width=0.75), color="black") +

ylab("Substrate temperature °C") +

ggtitle("A")

box.bts <- ggplot(Subs, aes(x = Species, y = Eye, fill = Microhabitat)) +

geom_boxplot(aes(color = Month), alpha = 0.9) + # Set color aesthetic here

scale_fill_manual(values = c("#006400", "#545454")) +

scale_color_manual(values = c("#654321", "#FF6666", "darkorange")) + # Use the same colors for the border

theme(axis.text.x = element_text(face = "italic"),

axis.text = element_text(size = 14),

axis.title = element_text(size = 16, face = "bold"),

panel.background = element_blank(),

panel.grid.major = element_line(color = "gray90"),

panel.grid.minor = element_line(color = "gray90"),

axis.line = element_line(color = "black"),

title = element_text(size = 16)) +

stat_summary(fun.y="mean", geom="point", size=2,

position=position_dodge(width=0.75), color="black") +

ylab("Body temperature °C") +

ggtitle("C")

box.Fs <- ggplot(Subs, aes(x = Species, y = Fluxometer, fill = Microhabitat)) +

geom_boxplot(aes(color = Month), alpha = 0.9) + # Set color aesthetic here

scale_fill_manual(values = c("#006400", "#545454")) +

scale_color_manual(values = c("#654321", "#FF6666", "darkorange")) + # Use the same colors for the border

theme(axis.text.x = element_text(face = "italic"),

axis.text = element_text(size = 14),

axis.title = element_text(size = 16, face = "bold"),

panel.background = element_blank(),

panel.grid.major = element_line(color = "gray90"),

panel.grid.minor = element_line(color = "gray90"),

axis.line = element_line(color = "black"),

title = element_text(size = 16)) +

stat_summary(fun.y="mean", geom="point", size=2,

position=position_dodge(width=0.75), color="black") +

ylab("Illuminace (lx)") +

ggtitle("D")

#### Relative humidity and microhabitats

fit.TLh <- lm.rrpp(TL.rb$EH ~ TL.rb$Month, na.rm = T)

anova(fit.TLh)

fit.LSh <- lm.rrpp(LS.hv$EH ~ LS.hv$Month, na.rm = T)

anova(fit.LSh)

box.hs <- ggplot(Subs, aes(x = Species, y = EH, fill = Microhabitat)) +

geom_boxplot(aes(color = Month), alpha = 0.9) + # Set color aesthetic here

scale_fill_manual(values = c("#006400", "#545454")) +

scale_color_manual(values = c("#654321", "#FF6666", "darkorange")) + # Use the same colors for the border

theme(axis.text.x = element_text(face = "italic"),

axis.text = element_text(size = 14),

axis.title = element_text(size = 16, face = "bold"),

panel.background = element_blank(),

panel.grid.major = element_line(color = "gray90"),

panel.grid.minor = element_line(color = "gray90"),

axis.line = element_line(color = "black"),

title = element_text(size = 16)) +

stat_summary(fun.y="mean", geom="point", size=2,

position=position_dodge(width=0.75), color="black") +

ylab("Relative humidity %") +

ggtitle("B")
