# Supplementary material for: Niche Differences in Coexisting Species: Ecological Insights Into the Role of Activity Patterns, Space Use, and Environmental Preferences
Source: Ecol Evol. 2025 Jul 31;15(8):e71802. doi: 10.1002/ece3.71802 (PMC12314190; doi:10.1002/ece3.71802)
Supplement: Supplementary file 3 — Appendix S3 [file ECE3-15-e71802-s001.docx]

# ---------------------------------------------

# R Script for Activity Pattern and Overlap Analysis

# Data source: CastroDB - Activity data of lizards by species and season

# Author: Carolina Reyes-Puig (2025)

# Description:

# This script performs activity pattern analysis using kernel density estimation

# and calculates temporal overlap between species and seasons. Includes

# bootstrapped confidence intervals and visualizations.

# ---------------------------------------------

library(activity)

library(overlap)

library(tidyverse)

### Format time and filter for adults only

CastroDB$Time <- format(as.POSIXlt(CastroDB$Time, format = "%H:%M"), format = "%H:%M")

CastroDB$Date <- format(as.Date(CastroDB$Date), format = "%Y-%m-%d")

A.DB <- CastroDB %>% filter(Status == "Adult")

### Convert time to radians for circular analysis

A.DB$TimeDecimal <- as.numeric(substr(A.DB$Time, 1, 2)) + as.numeric(substr(A.DB$Time, 4, 5)) / 60

A.DB$rads <- (A.DB$TimeDecimal / 24) * 2 * pi

### Bandwidth and kernel fitting per species

bw.sp <- tapply(A.DB$rads, A.DB$Species, function (x) { bwcalc(x, K = 3)} )

fit.act.sp <- tapply(A.DB$rads, A.DB$Species, function (x) { fitact(x, sample = "data", reps = 9999)} )

cmean.sp <- tapply(A.DB$rads, A.DB$Species, cmean)

fit.lacerta <- fit.act.sp$`L. schreiberi`

fit.timon <- fit.act.sp$`T. lepidus`

db.rds.lacerta <- A.DB[A.DB$Species %in% "L. schreiberi", ]

db.rds.timon <- A.DB[A.DB$Species %in% "T. lepidus", ]

### Plot activity density per species

par(mfrow = c(1, 2), cex.axis = 1.5, cex.lab = 1.5, cex.main = 1.5, cex.sub = 1.5, mar = c(5, 5, 4, 2) + 0.1)

densityPlot(db.rds.timon$rads, rug = F, col = "#00008B",extend="#CDC5BF", lwd = 3, main = "")

title(main = "A", adj = 0, line = -1.5, cex.main = 2)

densityPlot(db.rds.lacerta$rads, rug = F, col = "#7CCD7C",extend="#CDC5BF", lwd = 3, main = "")

title(main = "B", adj = 0, line = -1.5, cex.main = 2)

### Compare activity curves between species

c.cKern <- compareCkern(fit.lacerta, fit.timon, rep = 9999)

c.act <- compareAct(list(fit.lacerta, fit.timon))

### Activity and season

##Lacerta

db.rds.lacerta.sp <- db.rds.lacerta[db.rds.lacerta$Season %in% "Spring ", ]

db.rds.lacerta.sm <- db.rds.lacerta[db.rds.lacerta$Season %in% "Summer ", ]

par(mfrow = c(1, 2),

cex.axis = 1.5,

cex.lab = 1.5,

cex.main = 1.5,

cex.sub = 1.5,

mar = c(5, 5, 4, 2) + 0.1)

densityPlot(db.rds.lacerta.sp$rads, rug = F, col = "#7CCD7C",extend="#CDC5BF", lwd = 3, main = "")

title(main = "A", sub = NULL, adj = 0, line = -1.5, cex.main = 2)

abline(v = c(6.3, 19+40/60), lty = 3, col = "black", lwd = 1.5)

densityPlot(db.rds.lacerta.sm$rads, rug = F, col = "#7CCD7C",extend="#CDC5BF", lwd = 3, main = "")

title(main = "B", sub = NULL, adj = 0, line = -1.5, cex.main = 2)

abline(v = c(6, 20+30/60), lty = 3, col = "black", lwd = 1.5)

##Timon

db.rds.timon.wi <- db.rds.timon[db.rds.timon$Season %in% "Winter ", ]

db.rds.timon.sp <- db.rds.timon[db.rds.timon$Season %in% "Spring ", ]

db.rds.timon.sm <- db.rds.timon[db.rds.timon$Season %in% "Summer ", ]

par(mfrow = c(1, 3),

cex.axis = 2,

cex.lab = 2,

cex.main = 2,

cex.sub = 2,

mar = c(5, 5, 4, 2) + 0.1)

densityPlot(db.rds.timon.wi$rads, rug = F, col = "#00008B",extend="#CDC5BF", lwd = 3, main = "")

title(main = "A", sub = NULL, adj = 0, line = -1.5, cex.main = 2.5)

abline(v = c(7, 17+45/60), lty = 3, col = "black", lwd = 2)

densityPlot(db.rds.timon.sp$rads, rug = F, col = "#00008B",extend="#CDC5BF", lwd = 3, main = "")

title(main = "B", sub = NULL, adj = 0, line = -1.5, cex.main = 2.5)

abline(v = c(6.3, 19+40/60), lty = 3, col = "black", lwd = 2)

densityPlot(db.rds.timon.sm$rads, rug = F, col = "#00008B",extend="#CDC5BF", lwd = 3, main = "")

title(main = "C", sub = NULL, adj = 0, line = -1.5, cex.main = 2.5)

abline(v = c(6, 20+30/60), lty = 3, col = "black", lwd = 2)

#### Overlapping estimates

overlap.est<- overlapEst(db.rds.lacerta$rads, db.rds.timon$rads, type="Dhat1")

overlap.est.sp <- overlapEst(db.rds.lacerta.sp$rads, db.rds.timon.sp$rads, type="Dhat1")

overlap.est.sm <- overlapEst(db.rds.lacerta.sm$rads, db.rds.timon.sm$rads, type="Dhat1")

boots <- bootstrap(db.rds.lacerta$rads, db.rds.timon$rads, 9999, type="Dhat1", cores=1)

boots.sp <- bootstrap(db.rds.lacerta.sp$rads, db.rds.timon.sp$rads, 9999, type="Dhat1", cores=1)

boots.sm <- bootstrap(db.rds.lacerta.sm$rads, db.rds.timon.sm$rads, 9999, type="Dhat1", cores=1)

mean(boots)

hist(boots)

bootCI(overlap.est, boots)

bootCI(overlap.est.sp, boots.sp)

bootCI(overlap.est.sm, boots.sm)

boot_result <- bootEst(db.rds.lacerta$rads, db.rds.timon$rads, type="Dhat1")

##compare between season

fit.lacerta.sp <- fitact(db.rds.lacerta.sp$rads, sample = "data", reps = 9999)

fit.lacerta.sm <- fitact(db.rds.lacerta.sm$rads, sample = "data", reps = 9999)

fit.timon.sp <- fitact(db.rds.timon.sp$rads, sample = "data", reps = 9999)

fit.timon.sm <- fitact(db.rds.timon.sm$rads, sample = "data", reps = 9999)

fit.timon.wi <- fitact(db.rds.timon.wi$rads, sample = "data", reps = 9999)

c.cKern.sp <- compareCkern(fit.lacerta.sp, fit.timon.sp, rep = 9999)

c.cKern.sm <- compareCkern(fit.lacerta.sm, fit.timon.sm, rep = 9999)

c.cKern.sp.timon <- compareCkern(fit.timon.sp, fit.timon.sm, rep = 9999)

c.cKern.wisp.timon <- compareCkern(fit.timon.wi, fit.timon.sp, rep = 9999)

c.cKern.wism.timon <- compareCkern(fit.timon.wi, fit.timon.sm, rep = 9999)

c.cKern.sp.lacerta <- compareCkern(fit.lacerta.sp, fit.lacerta.sm, rep = 9999)

compareAct(list(fit.lacerta.sp, fit.lacerta.sm))

compareAct(list(fit.timon.sp, fit.timon.wi))

compareAct(list(fit.timon.sp, fit.timon.sm))

compareAct(list(fit.timon.wi, fit.timon.sm))

par(mfrow = c(1, 3),

cex.axis = 2,

cex.lab = 2,

cex.main = 2,

cex.sub = 2,

mar = c(5, 5, 4, 2) + 0.1)

overlapPlot(db.rds.timon$rads, db.rds.lacerta$rads, main=" ", extend="#CDC5BF", rug = F, linecol = c("#00008B", "#7CCD7C"), linewidth = c(2, 2),

olapcol = "#708090", linetype = c(1, 1)) # general overlap

title(main = "C", sub = NULL, adj = 0, line = -1.5, cex.main = 2.5)

overlapPlot(db.rds.timon.sp$rads, db.rds.lacerta.sp$rads, main=" ", extend="#CDC5BF", rug = F, linecol = c("#00008B", "#7CCD7C"), linewidth = c(2, 2),

olapcol = "#708090", linetype = c(1, 1)) # spring overlap

abline(v = c(6.3, 19+40/60), lty = 3, col = "black", lwd = 2)

title(main = "D", sub = NULL, adj = 0, line = -1.5, cex.main = 2.5)

overlapPlot(db.rds.timon.sm$rads, db.rds.lacerta.sm$rads, main=" ", extend="#CDC5BF", rug = F, linecol = c("#00008B", "#7CCD7C"), linewidth = c(2, 2),

olapcol = "#708090", linetype = c(1, 1))

abline(v = c(6, 20+30/60), lty = 3, col = "black", lwd = 2)

title(main = "E", sub = NULL, adj = 0, line = -1.5, cex.main = 2.5)
